# Efficient co-expression of bicistronic proteins in mesenchymal stem cells by development and optimization of a multifunctional plasmid

**DOI:** 10.1186/scrt56

**Published:** 2011-03-14

**Authors:** Christopher D Krause, Lara S Izotova, Gwangwen Ren, Zeng-Rong Yuan, Yufang Shi, Chiann-Chyi Chen, Yacov Ron, Sidney Pestka

**Affiliations:** 1 Department of Molecular Genetics, Microbiology, and Immunology, RWJMS-UMDNJ, 675 Hoes Lane West, Piscataway, NJ 08854, USA; 2 Pestka Biomedical Laboratories, 131 Ethel Road West, Suite 6, Piscataway, NJ 08854, USA

## Abstract

**Introduction:**

Local synthesis of interferon within B16 tumors mediates anti-tumor effects. Based on reports that stem cells are recruited to tumors, and because systemic administration of interferon causes dose-limiting undesirable side effects, we wanted to improve the anti-tumor effects of interferon while simultaneously minimizing its systemic side effects by employing mesenchymal stem cells (MSCs) as tumor-localized ectopic producers of interferon. Many vectors exist to fulfill this purpose, but their transfection efficiency and resulting expression levels vary considerably.

**Methods:**

To follow both the recruitment to tumors and the synthesis of interferon by MSCs, we designed a bicistronic vector system that permits fluorescent visualization of vector-transfected and interferon-producing MSCs. We used Mu-IFNαA cDNA as the first cistron and the cherry fluorescent protein cDNA as the second cistron, whose translation requires the internal ribosome entry sequence (IRES) from the encephalomyocarditis virus 5' untranslated region. Observing inconsistent expression of these cistrons in various vectors and cell lines, especially compared with a control plasmid pmaxGFP, we optimized the expression of this bicistronic message by mutating pcDNA3 to facilitate exchange of the promoter and polyadenylation segments controlling both the gene of interest and the eukaryotic antibiotic resistance gene as well as the eukaryotic antibiotic resistance gene itself, and effectively compare the effects of these exchanges, creating plasmid pc3.5.

**Results:**

Murine MSCs stably and ectopically expressing Mu-IFNαA inhibited the establishment of tumors in homogeneic C57/BL6 mice. Mu-IFNαA expressed from the bicistronic message is fully biologically active, but is expressed at only two-thirds of the level observed from a monocistronic message. Cap-dependent translation is threefold more efficient than IRES-driven translation in 293T, B16, and MSC cell lines. Both efficient expression and good transfection efficiency require strong expression of the gene of interest and a chimeric intron. High doses of Mu-IFNαA within tumors inhibited tumor establishment but may not inhibit tumor growth.

**Conclusions:**

Our modified vector and its derived plasmids will find use in stem cell therapeutics, gene expression, mRNA regulation, and transcription regulation. Local release of Mu-IFNαA within tumors may differently affect tumor establishment and tumor growth.

## Introduction

Many molecular biologists that perform experiments involving ectopically expressed proteins work with a number of plasmids. Some of these plasmids are well validated, while others are used with little knowledge on how they were synthesized or on their validated sequence. Furthermore, each plasmid is generally designed with a single purpose (for example, studying promoters, ectopic expression, virus generation, or exploring mRNA stability), and often exhibit significant variation in their transfection efficiency within a given cell line, and especially among various cell lines. To offset these variations, a diverse array of reagents have been developed to transfect cell lines with particular plasmids, or to transfect cell lines that strongly resist taking in or expressing exogenous DNA such as immune cell lines or primary cell lines.

Among these recalcitrant cell lines are embryonic or adult stem cells. The latter have fewer ethic complications and, with improvements in their directed programming, are enjoying widespread potential in basic, translational, and clinical research in the treatment of many physiological diseases, especially those in which organ, tissue, or immune cell rebuilding or replacement is a key requirement. Among the most widespread adult-derived stem cells used for this research are mesenchymal stem cells (MSCs). These cells are recruited to tissues by chemotactic signals (often mediated by chemokines) and perform key roles after being recruited [1]. MSCs play a structural role during the rebuilding of tissues, or differentiate into cells that perform infrastructural roles in the tissue or organ (for example, vasculature, inter-organ compartmentalization) [1-3]. They also help to locally suppress the immune system that may detect these substantial tissue changes as a result of an exogenous agent [4-7]. Finally, MSCs are easily extracted from animals or from humans, minimizing immunological complications arising from introducing engineered cells or tissues and facilitating their use for tissue therapy.

Ironically, tumors recruit MSCs for largely similar purposes. As tumor cells proliferate, they require tissue support and integration with the circulatory system to sustain the large mass of cells; they develop vasculature and stroma derived from MSCs to fulfill these needs [8]. Additionally, the recruited MSCs may promote the local immunosuppression of the immune system [4-7] that functions innately to inhibit tumor growth. Nevertheless, the recruitment of MSCs to tumors has been utilized by several groups to try to deliver anti-tumor agents locally to reduce both the overall dose as well as the concomitant side effects to the patient.

Type I interferons such as IFNα are known to have anti-tumor activity, based initially on their ability to slow the proliferation of tumorigenic and transformed cell lines. Our laboratory demonstrated that injection of B16 cells that ectopically express Mu-IFNαA fail to grow to become palpable tumors [9]. The same is true of B16 cells ectopically expressing Mu-IFNγ and Mu-IFNλ2 [10,11]; notably, these three interferons bind to distinct receptors, act under distinct situations, and exhibit distinct physiological functions. Additionally, the effects of interferon on tumor growth suppression are not restricted to the tumor cells themselves. Interferons act alone or can synergize with TNFα to inhibit proliferation of endothelial cells and angiogenesis [12-15], and in this way act to prevent tumor development. Type I interferons also influence the immune system: they enhance the activity of natural killer cells that possess anti-tumor activity [16-18], and they promote the upregulation of class I MHC complexes and alter peptide presentation in nonimmune cells to emphasize their detection by the adaptive immune system [19,20]. Interferons thus impede tumorigenesis by several distinct mechanisms.

The ectopic expression of type I interferons by MSCs results in considerably slowed tumor growth when these MSCs are injected into mice at the same time as injection of tumorigenic cells [21-26]. The reprogramming of tissues or the treatment of tumors can thus be accomplished by genetically engineered MSCs. However, for these MSCs to be useful, especially for a chronic disease like cancer, one must prove that MSCs are effectively targeted to the correct tissues, that the MSCs continue to produce their ectopic gene of interest (GOI), and that engineered MSCs persist in the host. Ideally, the expression of the GOI should also be restricted to the tissue that is being reprogrammed. To accommodate these quality-control requirements, one must select a proper promoter and a reporter that is easily detected in tissue sections or, if possible, *in vivo*. Also, implementation of useful 3' untranslated regions (UTRs) could further refine the expression.

To address all of these needs that require distinct genetic elements, we altered a well-validated plasmid (we chose plasmid pcDNA3, originally marketed by Invitrogen, Carlsbad, CA, USA) to facilitate the introduction of distinct genetic plasmid elements. We tested various derivatives of this plasmid in cell lines that are used for ectopic expression (human 293T cells), in murine models of aggressive melanoma (murine B16 melanoma cells), and in murine MSCs. By implementing an internal ribosome entry sequence (IRES) from encephalomyocarditis virus (EMCV), we tightly coupled the expression of the GOI and that of the reporter (in this case, the cherry fluorescent protein (ChFP)) by constructing a bicistronic mRNA to ensure that transcription of the GOI occurs. We found a surprising correlation of various elements in strong expression and effective transfection efficiency. The predominant effectors are the vector backbone and the strength of promoter driving the GOI, while minor effects were seen by altering the overall expression of the eukaryotic antibiotic resistance gene.

## Materials and methods

### Ethical approval

To perform the research within the present manuscript, our laboratory received approval from the Institutional Review Board of the Robert Wood Johnson Medical School under protocol 021996W0149 (approved 22 January 1996) 'Experimental Protocol for the Analysis of Serum from Interferon Cytokines, Lymphokines and Other Components' and under protocol 021995W0188 (approved 22 February 2009) 'Analysis of the Expression of Cytokines and their Receptor Chains: IFNγ, IFNα, IFNβ, IFNν, IL-4, IL-5, IL-6, IL-10, IL-12, IL-13, IL-18 and Related Class I and Class II Cytokines, and IL-10R1, IL-10R2, IFNγR1, IFNγR2, IFNαR1, IFNαR2 Receptors and Related Receptors JBP1, JBP2'.

To perform studies with live mice, we also received approval from the Institutional Animal Care and Use Committee at the Robert Wood Johnson Medical School under protocol I07-128-12 (approved 16 December 2009) 'Treatment of Malignancy in Mice Using Stem Cells for Delivery of Biotherapeutics'.

### Cell lines, media, and reagents

Human kidney epithelial 293T cells were obtained from ATCC (Manassas, VA, USA) and were grown in DMEM supplemented with 10% fetal bovine serum. Murine C57BL/6 B16F0 aggressive melanoma cells were grown in DMEM supplemented with 10% FCS. Monoclonal murine C57BL/6 bone-marrow-derived MSCs were obtained as previously described [8,10], and were grown in DMEM supplemented with 10% fetal bovine serum and 10 ng/ml human basic fibroblast growth factor, obtained from Peprotech (Rocky Hill, NJ, USA). FCS was obtained from Gemini BioProducts (Sacramento, CA, USA). Representative clones harboring bicistronic messages from stably transfected population were amplified by limiting dilution in 96-well dishes. Medium inside wells containing stem cells was changed every 3 to 4 days because basic fibroblast growth factor is labile at 37°C under our incubation conditions. Expanded clones were visualized by fluorescence microscopy to confirm the uniform expression of bicistronic messages throughout the population. Only expanded monoclonal populations are used in experiments with mice.

*Pyrococcus furiosus *(Pfu) DNA polymerase was obtained from Stratagene/Agilent, Inc. (Santa Clara, CA, USA). *Thermus aquaticus *(Taq) DNA polymerase, T4 DNA ligase, shrimp alkaline phosphatase, and all restriction enzymes were obtained from NEB (Ipswich, MA USA). All primers were obtained from Integrated DNA technologies, Inc. (Coralville, IA, USA) and were used without further purification other than desalting unless specified.

Murine IFNαA (purified, 1,350 units/μl - also known as Mu-IFNα3; #12100-1) as well as rat anti-mouse monoclonal neutralizing antibodies raised against Mu-IFNαA (#22100-1) were gifts from PBL Interferon Source (Piscataway, NJ, USA).

Polyethyleneimine (PEI) was obtained from Polysciences, Inc. (Warrington, PA, USA) as a desiccated linear molecule averaging 25 kDa molecular weight. Aqueous solutions of PEI (1 mg/ml) were prepared by dissolution in water, and titration with hydrochloric acid until the solution clears. After 1 hour, the solution is neutralized to pH 7.1 with sodium hydroxide. After filtration, aliquots were stored at -70°C until use. Metafectene and Metafectene Easy were obtained from Biontex GmbH (Planegg/Martinsreid, Germany). Fugene-6 and Fugene HD were obtained from Roche Diagnostics (Indianapolis, IN, USA). The Amaxa Nucleofection Kit V was obtained from Amaxa/Lonza, Inc. (Gaithersburg, MD, USA).

### Site-directed mutagenesis of pcDNA3

We introduced several nucleotide mutations into the pcDNA3 backbone to create unique restriction sites in pcDNA3 that allow exchange of either the promoter-driving sequences within the multicloning site, the 5' UTR between the promoter and the multicloning site, the 3' UTR after the multicloning site, or the resistance gene itself. Plasmids were mutated largely following the protocol offered in the QuikChange site-directed mutagenesis kit (Stratagene/Agilent, Inc.). Briefly, a 25 μl reaction containing 2.5 μl of 10× Pfu polymerase buffer, 100 pmol forward primer and 100 pmol complementary (reverse) primer (each purified by PAGE to remove mis-synthesized primers that lower the efficiency of the protocol), 1 μl DMSO, 1 μl of 50 mM MgCl_2_, 0.5 μl of 50× dNTP mix (10 mM each dNTP), 30 ng plasmid, 0.5 μl (1.25 units) Pfu polymerase and water, was subjected to PCR (in a Techgene thermal cycler; Techne, Cambridge, UK) to amplify a mutated plasmid. After a 2-minute incubation at 95°C, the solution was subjected to 18 cycles of: 30 seconds at 94°C; 1 minute at 55°C; and 18 minutes at 68°C, followed by 10 minutes at 72°C and incubation at 4°C until used. Then 5 units of *Dpn*I restriction endonuclease was added to the reaction mix to digest the parental plasmid, and the reaction was incubated at 37°C for 90 minutes. Two 5 μl aliquots are removed and resolved by agarose gel electrophoresis before and after the restriction digest to ensure the PCR reaction and the restriction digest succeeded. After digestion, a 2 μl aliquot was transformed into *Escherichia coli *strain DH5α by chemical transformation. Plasmid DNA was isolated from well-isolated bacterial colonies and an aliquot of that DNA was characterized by restriction digest and/or sequencing reaction to confirm the mutagenesis. While describing the complementary primer pairs in this manuscript, only the sequence of the forward primer is shown, although both it and its reverse complement were used.

To create plasmid pc3.1, a *Cla*I site (which is not digested in *dam*^*+ *^*E. coli *strains) was inserted just before the cytomegalovirus (CMV) promoter. The forward primer used to mutate pcDNA3 is: CTGCTTCGCGATGTACGGG**ATC**GATATACGCGTTGACATTG. These oligonucleotides overlap nucleotides 201 to 241 of plasmid pcDNA3. Mutated nucleotides compared with pcDNA3 are set in boldface, and restriction sites (*Nru*I, *Cla*I, and *Mlu*I) are underlined.

To create plasmid pc3.2, *Afl*II and *Hpa*I sites were inserted just 3' of the transcription start site and prior to the T7 primer sequence. The forward primer used to mutate pc3.1 is: CTAGAGAACCCACTGCTTA**AG**GG**G**TTA**A**CGAAATTAATACGACTCAC. These oligonucleotides overlap nucleotides 829 to 875 of plasmid pcDNA3. Mutated nucleotides compared with pcDNA3 are set in boldface, and restriction sites (*Afl*II and *Hpa*I) are underlined.

To create plasmid pc3.3, *Age*I, *Sac*II, and *BsiW*I sites were inserted just after the bovine growth hormone polyadenylation signal and a *Pvu*II site was concomitantly removed. The forward primer used to mutate pc3.2 is: CTGAGGCGGAAAGAACC-GC**G**GG**C**G**TA**C**G**A**ACC**GGTATCCCCACGCGCCC. The dash in the primers indicates that we intentionally deleted one nucleotide from plasmid pc3.2 where a P*vu*II site laid. These oligonucleotides overlap nucleotides 1,270 to 1,319 of plasmid pcDNA3. Mutated nucleotides compared with pcDNA3 are set in boldface, and restriction sites (*Age*I, *Sac*II, and *BsiW*I) are underlined.

To create plasmid pc3.4, a *BssH*II site was inserted just prior to the simian virus-40 (SV40) promoter that drives the neomycin resistance gene. The forward primer used to mutate pc3.3 is: CTGATTTAACAAAAATTTA**G**CGCG**C**ATTAATTCTGTGGAATGTG. These oligonucleotides overlap nucleotides 1,713 to 1,756 of plasmid pcDNA3. Mutated nucleotides compared with pcDNA3 are set in boldface, and the *BssH*II restriction site is underlined.

We created plasmid pc3.5 from plasmid pc3.3 in a single step by employing in a single reaction the primer pair used to generate plasmid pc3.4 and a second pair of primers that inserted a nonmethylated *Cla*I site before the SV40 polyadenylation motif and removed an *Nae*I site. This second forward primer is: GGAATCGTTTTCCGGGAC**AT**CG**AT**TGGATGATCCTCCAGCGC. These oligonucleotides overlap nucleotides 3,044 to 3,085 of plasmid pcDNA3. Mutated nucleotides compared with pcDNA3 are set in boldface, and restriction sites (*Cla*I) are underlined.

**Figure 1:**
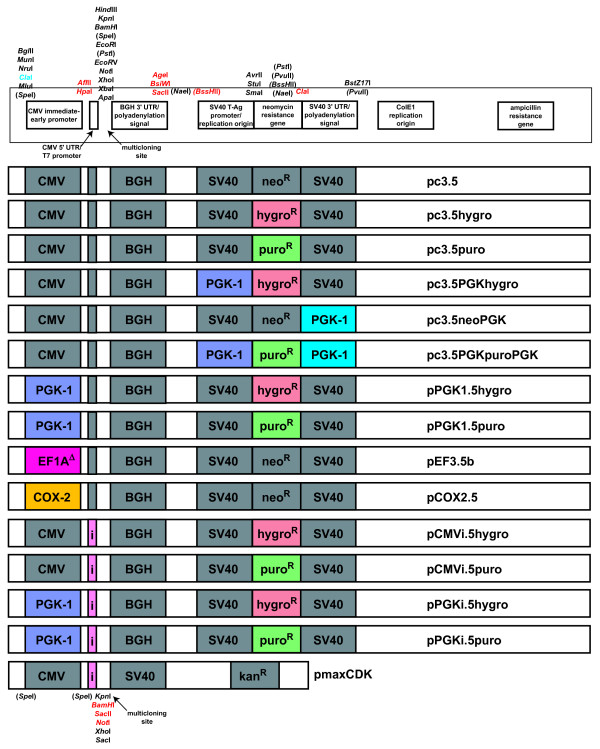
**Schematic of plasmids pc3.5 and pmaxCDK**. Unique restriction sites bordering various genetic elements of the plasmid are shown for pc3.5 (top) or for pmaxCDK (bottom). Various defined genetic elements such as promoters, polyadenylation sequences, and antibiotic resistance elements are labeled. Restriction sites bordering important eukaryotic genetic elements are shown. Sites within parentheses are not unique within pc3.5 but become unique upon the following exchanges: neomycin ORF to either hygromycin or puromycin ORF (*BssH*II, *Pvu*II and *Nae*I), cytomegalovirus (CMV) promoter to the EF1A or cyclooxygenase (COX)-2 promoter (*Spe*I), and neomycin ORF to puromycin ORF (*Pst*I). Restriction sites set in red were introduced; the cryptic introduced *Cla*I site is set in blue. Middle: each plasmid used in this manuscript is shown, with colored and labeled squares denoting a sequence that was introduced. Elements introduced include the human phosphoglycerate kinase (PGK)-1 genomic promoter (in purple), a truncated human elongation factor-1α (EF1A) promoter (in red, see Figure 6), the human COX-2 genomic promoter (in yellow), the β-globin/IgG synthetic chimeric intron (in pink), the hygromycin resistance gene (in orange), the puromycin ORF (in green), and the human PGK-1 polyadenylation sequence (in blue). BGH, bovine growth hormone; SV40, simian virus-40.

Control experiments demonstrated that plasmid pc3.5 expresses proteins as well as pcDNA3 in 293T cells and CHO cells, confers neomycin resistance to transfected mammalian cell lines equivalent to that of pcDNA3, and yielded as much plasmid from upon isolation from *E. coli *as plasmid pcDNA3 (data not shown). A schematic of the multifunctional plasmid pc3.5 is shown in Figure 1.

### Polymerase chain reaction

Nearly all of the elements used in the present manuscript were generated by PCR from either human genomic DNA (isolated from 293T cells using Stratagene's large-scale genomic DNA isolation kit) or from various plasmids. Either Taq or Pfu DNA polymerase were used. Typically, reactions had a final volume of 50 to 100 μl and consisted of (in final concentrations) the matched polymerase buffer, 500 μM each dNTP, 2 mM MgCl_2_, 10 pmol forward and 10 pmol reverse primers, either 5 ng plasmid or 1 μg genomic DNA, and 2.5 units polymerase. The amplification of the product was done with the following conditions: 2 minutes at 95°C and, 30 cycles of: 45 seconds at 94°C; 45 seconds at 5 to 10°C below the lowest T_m _of the primer pair; and 1 minute/kbp (rounded up to the next higher minute) target length at 68°C (for Pfu polymerase) or 72°C (for Taq polymerase), a final extension at 72°C for 10 minutes, and incubation at 4°C until needed. PCR products were cleaned of polymerase, buffer, primers and dNTPs with the QIAQuick PCR purification kit (Qiagen, Inc., Valencia, CA, USA). After their integration into a plasmid vector, the entire PCR product was sequenced within the plasmid using external primers to confirm sequence integrity. The details of each PCR product and the primers used to amplify templates to create them are described in Supplementary Text S1 in Additional file 1.

### Vector construction

After digestion, all vector and insert fragments are resolved by agarose gel (0.8 to 1.2% v:v) electrophoresis, then purified by the QIAQuick gel extraction kit (Qiagen, Inc.). Ligations were done with up to 200 ng total vector and insert in a total volume of 10 or 20 μl, depending on whether a standard ligation with T4 DNA Ligase or a Quick Ligase kit (both from New England Biolabs, Inc.) were done. After ligation, an aliquot of the mixture was transformed into chemically competent *E. coli *DH5α cells prepared in-house by an established protocol [27]. Well-isolated individual colonies were amplified to harvest plasmid DNA, whose overall sequence was verified by restriction digest and by sequencing of PCR-derived inserts. The details of each plasmid synthesized are described in Supplementary Text S2 in Additional file 2; most of these plasmids are depicted schematically in Figure 1.

### Transfection and antibiotic selection

Cells were transfected with PEI using modifications of several established protocols. The evening before transfection, cells were plated (typically in six-well dishes) at a density so that they were at 50 to 80% confluence when transfected. On the day of transfection, 2 μg DNA were diluted into 250 μl DMEM without FCS, and an optimal amount of PEI (usually 2 to 5 μg in water) was added to the diluted DNA. After 15 minutes of incubation at room temperature, the mixture was added to cells dropwise after removal of the conditioned medium, with swirling of the medium to distribute the reagent. Immediately afterwards, 2 ml complete medium was added. Expression of genes was detectable after 12 to 24 hours and was optimal after 24 to 48 hours.

Cells were transfected with Metafectene Pro or Metafectene Easy using the protocol suggested by the manufacturer with only minor modifications. Briefly, 2 μg DNA was mixed with 2.5 μl Metafectene Easy (or 3 μl Metafectene Pro) prediluted into 50 μl EASY buffer made from a 10× concentrate diluted with water. After 15 minutes of incubation at room temperature, the lipocomplexes were added to cells to which 2 ml fresh complete medium was added. Expression was gauged after 48 hours.

Cells were transfected with Fugene (Roche Diagnostics) or with the Amaxa/Lonza Inc. nucleofection system according to the manufacturers' instructions.

We used an identical protocol for stable transfection as for transient transfections in MSCs and B16 cells, except that on the third day geneticin sulfate (1 mg/ml), hygromycin sulfate (50 μg/ml for stem cells, 100 μg/ml for B16 cells), or puromycin sulfate (25 μg/ml) was added to the complete medium. Selection was continued until most cells in the population had died (10 days for G418 and 5 to 7 days for puromycin and hygromycin) and colonies of antibiotic-resistant cells are seen.

### Interferon assays

The titer of Mu-IFNαA released by mMSCs in conditioned medium was gauged by an assay that determines biologically active interferon, and by an assay that detects immunoreactive Mu-IFNαA. The anticytopathic effect inhibition assay (the antiviral assay) was performed on L929 cells as originally described [28]. An ELISA designed to detect immunoreactive Mu-IFNα (#42100-1; PBL Interferon Source) was performed according to the manufacturer's instructions.

### Fluorescence-activated cell sorting

Flow cytometry was performed with a Becton-Dickinson FACScan (Franklin Lakes, NJ, USA). The FL1 (530/30 nm barrier emission filter) and FL3 (650 long-pass emission filter) channels were compensated against the FL2 (585/42 barrier emission filter) channel prior to data collection to minimize the contribution of EGFP fluorescence in the FL3 channel and the contribution of ChFP fluorescence into the FL1 channel. A primary gate on a forward scatter:side scatter dotplot was used to isolate intact cells. An inverse gate on an FL1:FL3 dotplot was applied to mask cells that do not contain significant levels of GFP or ChFP fluorescence, identified by the dispersion of cells that were transfected with plasmids encoding no fluorescent proteins. The ungated cells (possessing significant GFP-based or ChFP-based fluorescence) were then analyzed for their fluorescence. Data were collected and processed with the CellQuest 3.3 software package (Becton-Dickinson). Histograms and dotplots were processed with WinMDI2.9 (Joseph Trotter, The Scripps Research Institute, La Jolla, CA, USA) for presentation in this manuscript.

### Mice, stem cell injection and tumor growth monitoring

Two different protocols were used for these experiments. In both cases, C57BL/6 mice (initially from Jackson Laboratories for the first experiment, but subsequently from the National Institutes of Health for the second experiment) were housed in standard housing and were nourished liberally. Mice were housed for 7 days prior to the injection of cells.

In the first experiment, B16 melanoma cells (1 million cells/limb) or PBS carrier were injected subcutaneously into each hind limb (2 million total cells per mouse) on day 0. On days 0, 3, and 6, 500,000 MSCs (or PBS carrier) were injected into the tail vein of each mouse. When tumors became palpable at 7 days, one group of mice was injected at days 7, 10 and 13. The maximum diameter of each hind limb was measured at days 9 and 13, and the days of death for each mouse were noted. Statistical analysis of viability curves was performed with the log-rank test method. Statistical comparisons of tumor diameters between groups of mice were done with the unpaired two-tailed *p *test.

An alternative protocol was used for the second experiment. Here, 100,000 B16 cells were injected subcutaneously into the right flank; at days 0, 3, and 6, 500,000 MSCs were injected into the tail vein of each mouse (three mice in six groups; one group for each clonal stem cell line). The growth of each tumor at the injection site was monitored over a 35-day period. Mice harboring tumors over 25 mm × 25 mm (lateral tumor area) were sacrificed. The dorsoventral and rostrocaudal tumor diameters of surviving mice were measured every 2 to 3 days after the tumor became palpable (after 10 to 20 days).

## Results and discussion

### Type I interferon secretion by stem cells slows tumor growth in mice

We first wanted to establish that we could inhibit tumor growth with MSC-synthesized interferon using our mouse models. Because IFNβ is known to have strong immunosuppressive activity (hence its use in treating multiple sclerosis), and may inhibit an innate anti-tumor immune response, we chose to instead utilize Mu-IFNαA to test the effect of a type I interferon more skewed to anti-tumor activities and less skewed to immunosuppression. We subcloned the Mu-IFNαA cDNA from plasmid pLNCX-Mu-IFNαA into plasmid pEF3 [29], and transfected the resultant plasmid pEF3-MuIFNαA into MSCs. After selection by challenge with G418, various subclones were tested for their ability to secrete bioactive Mu-IFNαA by screening IFNα secretion by antiviral assay and by ELISA (see below). A representative clone secreting a high dose of Mu-IFNαA was amplified and injected into C57Bl/6 mice either concurrently with B16 melanoma cells (resembling scenarios in which tumor development is predicted clinically or where a primary but potentially metastatic tumor was excised, and subordinate suppressed tumors may remain) or after palpable tumors were detected (modeling a scenario where a tumor is detected but tumor excision is infeasible or is being avoided), or was injected in the absence of B16 cells to ensure that these cells by themselves are not toxic to mice. As controls, B16 cells were injected in the absence of MSCs to follow how fast unencumbered tumors grow in mice; B16 cells were also co-injected with untransfected MSCs to ensure that the benefit of MSCs requires IFNαA secretion. The results of this experiment are described in Supplementary Text S3 in Additional file 3 and the data are shown in Supplementary Figure S1 in Additional file 4. Briefly, while initial growth of tumors is inhibited by IFNαA secreted by MSCs, suppression of established tumors by IFNαA was statistically ineffective.

Nevertheless, these results were promising enough to necessitate developing diagnostic tools to ease the identification (and perhaps permit noninvasive detection) of engineered MSCs during cloning and subsequent therapy. We rationalized the development of these tools for the following reasons. First, there was tremendous clonal variation in the amount of Mu-IFNαA released within a transfected population (CDK and LSI, unpublished observations). Because these cells grew very quickly, because single-cell suspensions were hard to obtain, and because our assay systems cannot easily determine whether all cells in the population express Mu-IFNαA, we could not easily and conclusively confirm that our clones were in fact monoclonal. Furthermore, we could not confirm whether the MSCs we introduced into mice survived for more than a few days. We do not know how many stem cells went to each tumor, whether the MSCs remain there during tumor development, or whether the engineered MSCs still express Mu-IFNαA. Additionally, aside from the detecting the gene of interest or the antibiotic resistance element, there is no way to immunologically or histologically distinguish engineered MSCs from MSCs that endogenously reside within the host, especially under therapeutic scenarios.

To address these issues we needed transfected cells that express not only Mu-IFNαA but also the monomeric ChFP, which folds quickly and emits red light upon illumination with blue or especially green or orange light. There are several ways to co-express two proteins ectopically in cells. Co-transfection of two distinct plasmids does not give a reliable indicator of IFNαA secretion given an amount ChFP fluorescence, especially from clone to clone of a transfected population (CDK, unpublished observations). Fusion of the two transcription units into a single plasmid to create a tandem vector did not improve this relation. The easiest way to assure the equivalent transcription of both ORFs, such that both ORFs are transcribed if the translation of one of the two ORFs is demonstrated, was to separate the two ORFs with an IRES.

### Construction of a bicistronic vector to monitor the expression level of a transgene

**Figure 2:**
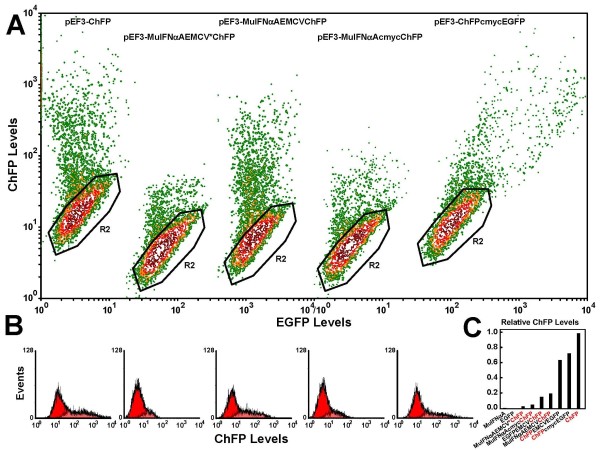
**Translation of cistrons from pEF3-based plasmids**. **(a) **293T cells were transfected using polyethyleneimine (PEI) with the following plasmids (from left to right): pEF3-ChFP, pEF3-MuIFNαAEMCV*ChFP, pEF3-MuIFNαAEMCVChFP, pEF3-MuIFNαAcmycChFP, and pEF3-ChFPcmycEGFP. The monomeric cherry fluorescent protein (ChFP)-expressing cells were resolved from nonfluorescent cells (isolated by the black gate labeled R2) by a contoured dotplot of FL1 (EGFP levels, horizontal) versus FL3 (ChFP levels, vertical) fluorescence. **(b) **Histograms of FL3 fluorescence of the ungated population (red) and the inversely-gated population indicating the ChFP-positive cells (light red, overlaid) are placed under the respective contour plots. **(c) **Comparison of ChFP translation rates. The mean red fluorescence of the inversely gated cells are shown for cells transfected with the following plasmids (from left to right): no ChFP cistron (pEF3-MuIFNαA, pEF3-EGFP), ChFP as the second cistron (pEF3-MuIFNαAEMCV*ChFP, MuIFNαAcmycChFP, pEF3-MuIFNαAEMCVChFP, pEF3-EGFPEMCVChFP), ChFP as the first cistron (pEF3-ChFPEMCVEGFP, pEF3-ChFPcmycEGFP), and ChFP as the only cistron (pEF3-ChFP).

Our first construction placed the Mu-IFNαA cDNA after the elongation factor-1α (*EF1A*) promoter and placed the ChFP cDNA after the IRES encoded by the encephalomyocarditis virus (EMCV) genome, so that translation of Mu-IFNαA is cap-dependent while translation of ChFP is IRES-dependent. We observed that, when expressed as a monocistronic message (from plasmid pEF3-ChFP; Figure 2a,b left), translation of the ChFP was about 30-fold stronger than in our first bicistronic plasmid pEF3-MuIFNαAEMCV*ChFP (Figure 2a,b second from left).

Sequence analysis revealed that this EMCV IRES was mutated and its efficiency inhibited by sevenfold to 10-fold (Figure 2). This mutation eliminated a *Kpn*I site within the IRES that lies along an important secondary structure element [30-33]. We instead used a sequence that more closely resembled the wildtype viral genome sequence (only differing in the mutation of an *ApaL*I site and the deletion of one A in a seven-A stretch, both present in the source plasmid). When we utilized this EMCV IRES (to create plasmid pEF3-MuIFNαAEMCVChFP), the translation of ChFP was much more efficient (about 16 to 20% that of the monocistronic message; Figure 2a,b middle).

We wanted to test an IRES of cellular origin that is resistant to inhibition of cap-dependent protein synthesis [34-37] or interferon effects [38] - that from the c-myc P2 mRNA [39,40]. c-Myc-dependent ChFP translation in plasmid pEF3-MuIFNαAcmycChFP, however, was poor (about 5%; Figure 2a,b second from right). Additionally, we found that translation of the c-myc IRES-dependent cistron was not uniform with respect to the translation of EGFP (Figure 2a right); this was explained by the known ability of the c-myc IRES to allow translation under conditions when cap-dependent protein synthesis is inhibited, such as mitosis (Supplementary Figure S2, left in Additional file 5), genotoxic stress or apoptosis (Supplementary Figure S2, right in Additional file 5) [35,41]. Because of these peculiarities and because the EMCV IRES was more well defined structurally and produced sufficient levels of ChFP for our purposes, we restricted our subsequent work to the optimal EMCV IRES.

Irrespective of which IRES was used, translation of ChFP from the first cistron was about 65 to 70% as efficient as that translated from a monocistronic message (Figure 2c, compare the rightmost three lanes), or about three to 3.5 times as efficient as translation from EMCV IRES-controlled cistron (Figure 2c, compare lanes 5 to 7).

**Figure 3:**
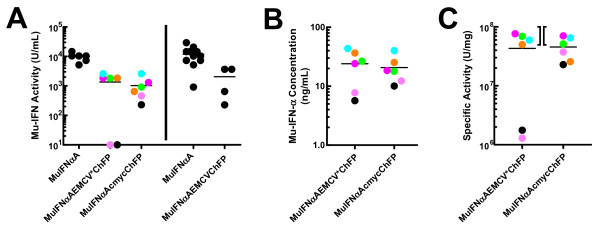
**Bioactivity of Mu-IFNαA translated from bicistronic vectors**. In conditioned medium from mesenchymal stem cell (MSC) clones where both bioactivity and immunoreactivity were measured, the specific activity of interferon in each medium was calculated and each clone was separately color-coded. In all cases, the activity was determined by comparison with a standard murine interferon, produced in *Escherichia coli *and purified to homogeneity, and the mean of each group of values are shown with a horizontal line. **(a) **Left: conditioned medium from six monoclonal MSC lines stably transfected with (from left to right) pEF3-MuIFNαA, pEF3-MuIFNαAEMCV*ChFP and pEF3-MuIFNαAcmycChFP were assayed for bioactivity by the antiviral assay. Right: in a separate experiment, conditioned medium from 12 monoclonal MSC lines stably transfected with pEF3-MuIFNαA and from four monoclonal MSC lines stably transfected with pEF3-MuIFNαAEMCVChFP were assayed. **(b) **Conditioned medium from six monoclonal MSC lines stably transfected with pEF3-MuIFNαAEMCV*ChFP (left) and pEF3-MuIFNαAcmycChFP (right) were assayed for Mu-IFNα immunoconcentration by ELISA. **(c) **Specific activities of the Mu-IFNαA within each conditioned medium were calculated by dividing the bioactivity by the immunoconcentration. Bacterial recombinant Mu-IFNαA purified to homogeneity has a specific activity of 5 × 10^7 ^to 10 × 10^7 ^units/mg; this range is indicated with the brackets between the two datasets. ChFP, monomeric cherry fluorescent protein.

Although we know the both the IRES-dependent and the cap-dependent cistrons are translated in our vector system (Figure 1), we wanted to prove that bioactive Mu-IFNαA was produced in our bicistronic system. To prove IFNαA was translated and secreted in an active form, plasmids pEF3-IFNαAEMCV*ChFP, pEF3-IFNαAcmycChFP and pEF3-IFNαAEMCVChFP were stably transfected in MSCs and several clonal cell lines from each population were isolated that demonstrated significant red fluorescence (it is more economical and faster to isolate clones by fluorescence than by interferon bioassay). Conditioned medium (2 ml medium collected after 2 days) from these monoclonal cell lines as well as from several MSC monoclonal cell lines expressing plasmid pEF3-MuIFNαA (one of which was used in Supplementary Figure S1 in Additional file 4) all possessed bioactive IFNα (Figure 3a). Notably, conditioned medium from MSCs expressing Mu-IFNαA as the only cistron contained generally fivefold to eightfold more bioactive interferon than did conditioned medium from MSCs transfected with plasmids expressing bicistronic messages. Conditioned medium from MSC clones stably transfected with plasmids pEF3-IFNαAEMCV*ChFP and pEF3-IFNαAcmycChFP were tested by ELISA; all clones were found to produce protein that was immunoreactive with serum raised against murine IFNα (Figure 3b). To prove that the IFNαA released from these cell lines was completely active, we calculated the specific activity of the interferon by dividing the bioactivity by the immunoconcentration for the 12 clones. Ignoring clones that poorly express Mu-IFNαA (usually resulting in weak antiviral activity in conditioned medium), the clones secrete Mu-IFNαA with a specific activity of 2 × 10^7 ^to 8 × 10^7 ^units/mg, in good agreement with the published value of purified bacterial recombinant Mu-IFNαA (5 × 10^7 ^to 10 × 10^7 ^units/mg). We therefore conclude that fully bioactive Mu-IFNαA is released by these MSCs transfected with these vectors, and that both cistrons are translated.

**Figure 4:**
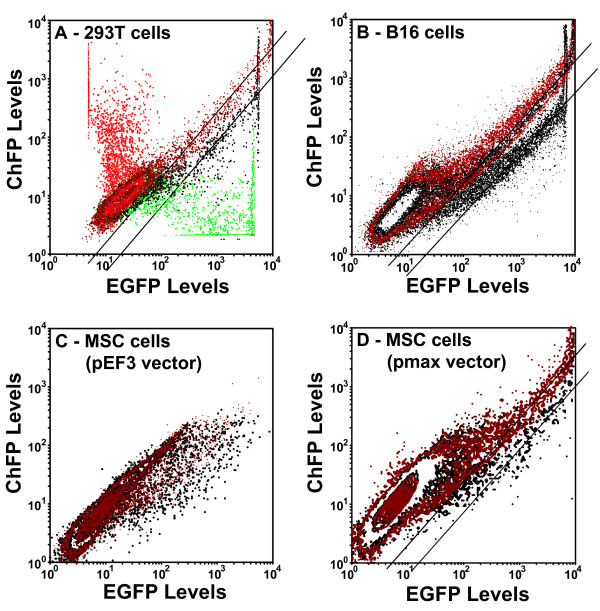
**Efficiency of cap-dependent versus internal ribosome entry site-dependent translation in various cell lines**. Plasmids pEF3-ChFP (in red), pEF3-EGFP (in green), pEF3-EGFPEMCVChFP (in black) and pEF3-ChFPEMCVEGFP (in brown) were transfected into **(a) **293T cells, **(b) **B16 cells and **(c) **mesenchymal stem cells (MSCs). Contour plots of FL1 (EGFP levels) versus FL3 (monomeric cherry fluorescent protein (ChFP) levels) were made of the green and red fluorescence from a sample of cells in each population. Lines were drawn through the medians of the cells demonstrating strong EGFP and ChFP fluorescence. **(d) **Because insufficient numbers of cells with strong fluorescence could be obtained in MSCs transfected with these plasmids, plasmids pmaxCDK-EGFPEMCVChFP (black) and pmaxCDK-ChFPEMCVEGFP (brown) were transfected into MSCs. Each population was translated in the dotplot so that the populations of nonfluorescent cells optimally overlapped.

Proving that both cistrons are expressed in our bicistronic plasmids, and that the first cistron is translated about three to 3.5 times better than the second cistron on a population level, we next sought to determine whether this difference in cistron expression applies to all cells in the population. To address this, we placed the EGFP cDNA after the *EF1A *promoter, and then made a construct in which we exchanged the places of ChFP and EGFP, creating plasmids pEF3-EGFPEMCVChFP and pEF3-ChFPEMCVEGFP. As shown in Figure 4a, while cells expressing either pEF3-ChFP or pEF3-EGFP express only one fluorescent protein and (after compensation of the green and red channels) have little color in the other channel, cells expressing plasmids pEF3-EGFPEMCVChFP and pEF3-ChFPEMCVEGFP exhibited both red and green fluorescence.

After translating various FL1:FL3 dotplots so that untransfected cells optimally overlap, the cells with brighter EGFP and ChFP fluorescence lie along parallel distributions in the overlaid dotplots (diagonal lines, Figure 4). Because these dotplots are double-logarithmic plots, parallel lines infer proportional expression of the two cistrons, with only the total intensity varying considerably throughout the population. The axial distance between the two parallel lines denotes the difference in the expression of one cistron if the expression of the other cistron is constant, and defines the difference in efficiency of translation between the cap and the IRES in bicistronic messages. In both 293T cells (Figure 4a) and in B16 melanoma cells (Figure 4b), cap-dependent translation was about threefold more efficient than IRES-dependent translation using pEF3-based plasmids. This ratio correlated with the data from Figure 2c. We could not obtain a definitive number in MSCs using these plasmids (Figure 4c), mostly because these plasmids were poorly transfected (see below). The above-observed threefold difference, however, is also seen in MSCs when an optimal vector system was used (Figure 4d). Our data therefore suggest that the efficiency of IRES-dependent translation does not vary significantly among cell lines.

### Variation of transfection efficiency is vector-dependent

**Figure 5:**
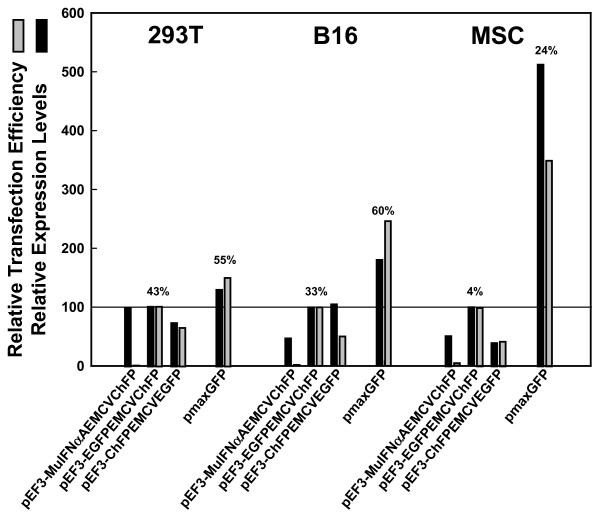
**Transfection efficiency of pEF3-based plasmids in 293T, B16 and mesenchymal stem cells**. Plasmids pEF3-MuIFNαAEMCVChFP, pEF3-EGFPEMCVChFP, pEF3-ChFPEMCVEGFP, and pmaxGFP were transfected (left) into 293T cells using polyethyleneimine, (middle) into B16 cells using Metafectene Easy and (right) into mesenchymal stem cells (MSCs) using Metafectene Easy. The percentage of cells containing significant levels of GFP and/or monomeric cherry fluorescent protein (ChFP) as well as the average green or red fluorescence of these cells were determined. These values were then divided by the related values determined in cells transfected with pEF3-EGFPEMCVChFP, whose actual transfection efficiency (as well as that from pmaxGFP) is shown above the bars. Relative transfection efficiency and relative average fluorescence intensity are indicated by black and grey bars, respectively. The results shown here are representative; the transfection efficiency as well as the relative expression or transfection difference between pEF3-based plasmids and pmaxGFP varied by as much as twofold depending on transfection reagent, passaging time, and cell density.

As we mentioned above, the transfection efficiency of our pEF3-based plasmids was rather poor in MSCs. In contrast, plasmid pmaxGFP (supplied as a positive control in the Amaxa/Lonza Inc. nucleofection kit) transfected only slightly better than our pEF3-based plasmids in 293T cells (Figure 5, left), but transfected more efficiently than pEF3-based plasmids in B16 cells (Figure 5, middle), and considerably better than our pEF3-based plasmids in MSCs (Figure 5, right). For reasons we do not understand, the transfection efficiency of plasmid pmaxGFP decreased less drastically in MSCs (from 55 to 60% down to 24%) relative to other cell lines than did the transfection efficiency of pEF3-based plasmids (from 43% down to 4%). This difference in transfection efficiency was not greatly affected by the transfection reagent whether less expensive reagents like PEI or Metafectene or more expensive optimized systems like the Amaxa/Lonza Inc. nucleofection system were used to warrant further use of expensive reagents. We therefore focused our studies with PEI, the reagent of choice with 293T and B16 cells, or with Metafectene Easy, the reagent of choice with MSCs and B16 cells.

To determine why pEF3-based plasmids transfected much more poorly than pmaxGFP in MSCs, we wanted to exchange various parts of pEF3 to optimize its expression, as the sequence of pmaxGFP is not publically available. Like most other expression vectors, however, pEF3-based plasmids only have useful restriction sites within the multicloning site that lie after the promoter that is driving expression of the GOI. Because few useful sites lie outside this region, exchanging antibiotic elements, promoters or untranslated regions to optimize the plasmid poses a problem. To overcome this problem, we mutated plasmid pcDNA3 (a well-defined and validated plasmid from which plasmid pEF3 derived [42]) so that one can easily exchange the promoter driving the GOI, the polyadenylation site for the GOI, the promoter driving the antibiotic resistance gene, the resistance gene itself, and the polyadenylation site for the antibiotic resistance gene. The method by which we mutated pcDNA3 to make pc3.5 was described in Materials and methods. After making this plasmid, we noticed that, outside the antibiotic resistance gene, the *BssH*II, *Nae*I (both before the SV40 promoter) and *Pvu*II (after the SV40 polyadenylation site) sites are unique and can also be used for cloning if necessary. In fact, exchanging the neomycin resistance gene for that of hygromycin and (by virtue of a fortuitous mutation obtained during PCR amplification) puromycin made these three sites unique. From plasmid pc3.5 we made a variety of vectors to test which elements promote efficient expression in MSCs and easily produce stably transfected clonal cell lines; these vectors are shown schematically in Figure 1.

### Modification of the eukaryotic antibiotic resistance gene

**Figure 6:**
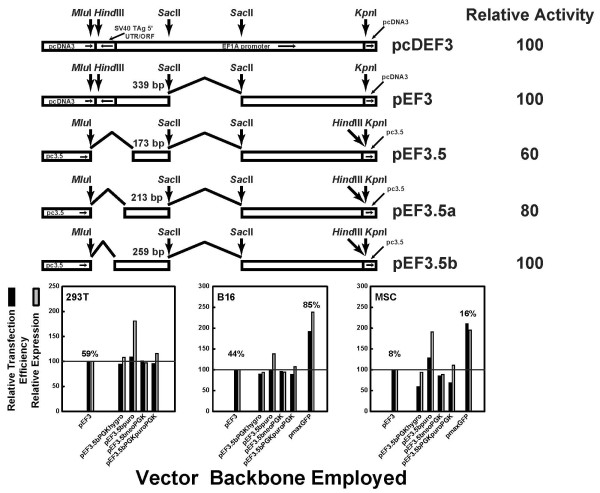
**Transfection efficiency of pEF3.5b-based plasmids harboring varying antibiotic resistance genetic elements**. Top: synthesis of truncated elongation factor-1α (EF1A) promoters. From the full-length EF1A promoter found in plasmid pcDEF3 [42], various truncations were performed to shorten the promoter without sacrificing activity. The uppermost truncation eliminates an unnecessary piece of the intron [50]. Truncations at the 5' end of the promoter to remove fragments derived from other vectors created vectors pEF3.5, pEF3.5a and pEF3.5b; only pEF3.5b expressed proteins as well as plasmids pEF3 and pcDEF3 (data not shown). Useful restriction sites within the promoter are shown. Truncations are shown with broken lines, and the amount of promoter upstream of the *Sac*II site is shown. The direction of each element in pcDEF3 is shown in the topmost schematic. Bottom: vectors pEF3, pEF3.5bPGKhygro, pEF3.5bpuro, pEF3.5bneoPGK, and pEF3.5bPGKpuroPGK, all harboring and expressing the bicistronic insert EGFPEMCVChFP, were transfected using polyethyleneimine into (left) 293T cells, (middle) B16 cells, and (right) mesenchymal stem cells (MSCs), and the percentage of successful EGFP expression (black bars) as well as the average fluorescence of the EGFP-expressing cells (gray bars) were reported as a percentage of that seen in cells transfected with pEF3-EGFPEMCVChFP. In B16 cells and in MSCs, plasmid pmaxGFP was also transfected to determine whether improved transfection in these cells was obtained. The actual percentage of transfection of pEF3-EGFPEMCVChFP and pmaxGFP is shown above their respective bars.

Our first hypothesis was that elements within the antibiotic resistance gene inhibited optimal expression of our bicistronic vector, as plasmid pmaxGFP has no eukaryotic resistance gene. In plasmid pc3.5, the SV40 promoter drives expression of the neomycin resistance gene; the SV40 polyadenylation site follows the neomycin resistance gene. We exchanged the SV40 promoter for the phosphoglycerate kinase-1 (PGK-1) promoter (driving the hygromycin resistance gene), replaced the neomycin resistance gene with the puromycin resistance gene, and utilized the PGK-1 polyadenylation site instead of the SV40 polyadenylation site to create plasmids pc3.5PGKhygro, pc3.5puro, and pc3.5neoPGK, respectively. We also exchanged all three elements to make plasmid pc3.5PGKpuroPGK. After placing three bicistronic GOIs (MuIFNαAEMCVChFP, EGFPEMCVChFP, and ChFPEMCVEGFP) under the control of a fully active 5' truncated EF3 promoter (Figure 6, top) in an intermediate plasmid, we recombined the EF3-driven GOIs with the pc3.5-derived plasmids with the modified antibiotic resistance genes. This resulted in plasmids pEF3.5bPGKhygro-(GOI), pEF3.5puro-(GOI), pEF3.5bneoPGK-(GOI), and plasmid pEF3.5bPGKpuroPGK-(GOI), as described in Materials and methods. Only the results for the bicistron EGFPEMCVChFP are shown.

We found that, except for the plasmids based on pc3.5puro, the expression of the GOIs did not vary among the plasmids in 293T cells (Figure 6, bottom left), implying that the elements controlling the antibiotic resistance gene do not greatly affect the expression of the GOI. Additionally, these changes did not improve the expression of pEF3.5b-based plasmids compared with pmaxGFP in either B16 cells (Figure 6, bottom middle), and may slightly inhibit the expression of the GOI in MSCs (Figure 6, bottom right). No exchange improved the transfection of pEF3.5b-based plasmids to that seen with pmaxGFP. Notably, the slightly improved expression of the GOI in the pEF3.5bpuro plasmid required the SV40 promoter and/or the SV40 polyadenylation site, as exchange of the SV40 elements for those of PGK1 to control the expression of the puromycin gene reduced the expression of the GOI. Nevertheless, we used hygromycin or puromycin in MSCs to more quickly select for resistant cell lines harboring the GOI and successfully amplify monoclonal MSC cell lines (data not shown). We therefore conclude that these elements could easily be exchanged to make plasmids with new properties that do not inhibit their ability to be transfected.

### Transfection efficiency is proportional to optimal expression of the mRNA of the GOI

We next hypothesized that elements controlling the expression of the GOI strongly influence the transfection efficiency of the resulting plasmid. We chose not to alter the polyadenylation sequences controlling the GOI because the bovine growth hormone polyadenylation sequence is already known to generate stable mRNA molecules and therefore promotes maximal expression of the GOI. Suspecting that the human *EF1A *promoter may not work well in these MSCs, we tried other promoters or implemented synthetic introns to more efficiently drive the GOI. First, we exchanged the CMV promoter in pc3.5hygro and pc3.5puro for the PGK-1 promoter, a promoter of similar strength but of cellular origin [43-46], to create plasmids pPGK1.5hygro and pPGK1.5puro.

The expression of a GOI is enhanced when introns are transcribed with the exons of a protein-coding RNA; because nascent RNAs that undergo splicing are more effectively coupled to the mRNA export machinery than are nascent RNAs that do not contain introns [47-49]. Indeed, the EF1A and polyubiquitin promoters are sixfold more active if the first intron within the 5' UTR is present [50,51]. We employed the mRNA export pathway by placing a synthetic and chimeric intron (composed of a β-globin splice site donor and an IgG splice site acceptor) within the 5' UTR between an intronless cDNA and either the CMV or the PGK promoters to make plasmids pCMVi.5hygro, pCMVi.5puro, pPGKi.5hygro and pPGKi.5puro. Finally, we tested a promoter (the human COX-2 promoter) that is inducible by inflammatory signals [52] and could further restrict the secretion of interferons to those MSCs that sense inflammatory signals, such as often found within tumors.

**Figure 7:**
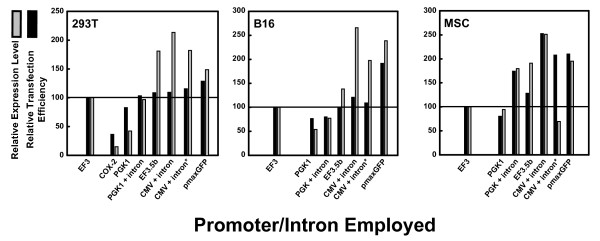
**Transfection efficiency of various pc3.5-based vectors harboring promoters and/or introns controlling the gene of interest**. Plasmids pEF3, pCOX2.5, pPGK1.5puro, pPGKi.5puro, pEF3.5bpuro, pCMVi.5puro, and pmaxCDK harboring the bicistronic insert EGFPEMCVChFP were expressed in (left) 293T cells, (middle) B16 cells and (right) mesenchymal stem cells (MSCs). These harbor the following promoters and/or introns: cyclooxygenase (COX)-2, phosphoglycerate kinase (PGK)-1, PGK-1 with β-globin/IgG chimeric intron, EF3.5b, cytomegalovirus (CMV) with β-globin/IgG chimeric intron, and CMV with β-globin/IgG chimeric intron. The asterisk implies expression of the insert from plasmid pmaxCDK instead of from plasmid pCMVi.5puro. Black bars, relative transfection efficiency compared with cells transfected with pEF3-EGFPEMCVChFP; grey bars, relative EGFP levels compared with cells transfected with pEF3-EGFPEMCVChFP. Plasmid pmaxGFP was also transfected to determine whether improved transfection in MSCs was obtained. Expression from the COX-2 promoter was insignificant and therefore not shown in B16 cells and in MSCs.

We found that the better transfection efficiency correlated with stronger average expression (Figure 7). This was true whether 293T cells (left), B16 cells (middle) or MSCs (right) were used, suggesting that both high transfection efficiency and good protein expression per cell are proportional to promoter strength. The CMV promoter with intron, whether in pc3.5-based plasmids or in pmax-based plasmids (Figure 7, with asterisk), generally gave both the highest expression and the highest transfection efficiency (between 150 and 250% that of pEF3-based plasmids). The synthetic intron, known to boost the activity of the CMV and SV40 promoters by up to eightfold in diverse studies, probably underlies the complementation of the sevenfold weakness of the CMV promoter relative to the EF1A promoter in our plasmid system. PGK promoters with included introns had activities comparable with those of EF1A promoters (between 70 and 130% that of pEF3-based plasmids). PGK-1 promoters without introns were one-half as strong as those with chimeric introns (with activities approximating 50 to 70% those of pEF3-based plasmids). The weakest of these promoters was the cyclooxygenase-2 promoter, with activities 15 to 40% those of EF3-based plasmids. The difference in promoters was less apparent in 293T cells than in B16 cells and in MSCs. Because we found that expression of our GOIs was largely equivalent under the CMV-intron promoter whether the backbone vector was pc3.5-based or whether it was pmaxGFP-based (Figure 7), we concluded that use of a sufficiently strong promoter is sufficient to enhance the expression of target genes in MSCs.

Expression of GFP from pmaxGFP was still better in most cases than expression of our GOIs, however, even when using the same vector and the same plasmid backbone. The GFPs used in our bicistronic vectors were designed to be nonaggregating and monomeric [53,54], whereas the primary sequence of GFP used in pmaxGFP closely resembles the wildtype aggregating GFP from the copepod *Chiridius poppei *[55]; the aggregates are sufficiently stable that they are toxic to cells. To test whether use of a nonaggregating mutant of this GFP modified the apparent expression efficiency of pmaxGFP, we substituted the wildtype GFP in plasmid pmaxGFP with its nonaggregating (but dimeric) mutant called TurboGFP [55] to create pmaxCDK-TurboGFP, and compared the expression of pmaxGFP with that of pmaxCDK-TurboGFP. We found that there was better transfection efficiency and overall expression of GFP from pmaxGFP than from pmaxCDK-TurboGFP, irrespective of transfection protocol or whether 293T cells, MSCs or B16 cells were used (Supplementary Figure S3 in Additional file 6).

### Auto-inhibitory actions of Mu-IFNαA on its own expression

**Figure 8:**
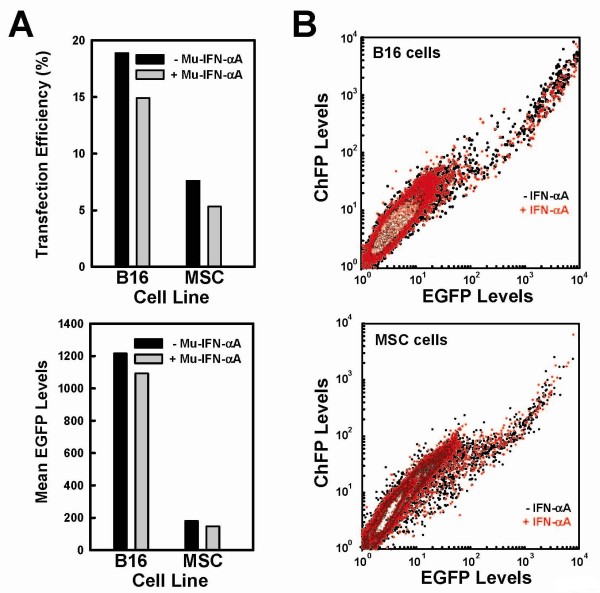
**Inhibition of transfection and expression by type I interferons in B16 and mesenchymal stem cells**. **(a) **B16 cells (left) and mesenchymal stem cells (MSCs) (right) were transfected with pmaxCDK-ChFPEMCVEGFP and were either not treated (black bar) or treated (grey bar) with 500 units/ml Mu-IFNαA. Top: bar graph depicting the transfection efficiency. Bottom: bar graph depicting the mean EGFP levels. **(b) **Contour plot of EGFP levels (horizontal axis) versus monomeric cherry fluorescent protein (ChFP) levels (vertical axis) in B16 cells (top) and in MSC cells (bottom). Cells not treated with Mu-IFNαA are black; cells treated with Mu-IFNαA are red.

We next wanted to understand why transfection of bicistronic messages encoding Mu-IFNαA was less efficient than those that do not encode Mu-IFNαA in murine (B16, MSC) cells but not in human (293T) cells (Figure 5). Because type I interferons are known to inhibit translation in a species-specific fashion, and because we suspected that lowered expression of proteins resulted in lower apparent transfection efficiencies, we hypothesized that the activity of murine type I interferon inhibits the expression and therefore the apparent transfection efficiency of plasmids encoding Mu-IFNαA in murine cells. To test this, and to test whether interferons differentially inhibit cap-dependent or EMCV IRES-dependent translation in our system, we transfected pmaxCDK-ChFPEMCVEGFP into B16 cells and MSCs and treated them with 500 units/ml Mu-IFNαA or with PBS alone. In both cell lines, both the transfection efficiency and the levels of EGFP decreased by 10 to 15% (Figure 8a). Co-transfection of pmaxCDK-ChFPEMCVEGFP with pEF3-MuIFNαA gave a slightly higher inhibition of transfection efficiency as well as decreased levels of fluorescent cells (data not shown). A comparison of the dispersion of fluorescent cells in the absence or presence of interferon demonstrated that there was a complete overlap in both cell lines (Figure 8b, black dots and red dots, respectively), implying that inhibition of expression did not favor cap-dependent over IRES-dependent translation. Conversely, treatment of B16 cells transfected with plasmid pCMVi.5puro-MuIFNαAEMCVChFP with neutralizing monoclonal antibodies specific to murine IFNα increased the apparent transfection efficiency as well as the mean ChFP levels by 10 to 15% (Supplementary Figure S4 in Additional file 7). This is consistent with the hypothesis that the presence of type I interferon in the medium inhibits transfection efficiency or expression levels. The transfection efficiency of this plasmid in MSCs was too poor for useful analysis to be done, even in the presence of monoclonal antibodies (data not shown).

### Dosage-dependent effects of MSC-synthesized Mu-IFNαA within B16 tumors in mice

Using derivatives of plasmid pc3.5, we created a panel of MSC lines with a wide variety of Mu-IFNαA secretion rates. These clones can be used to produce a dose-response curve *in vivo *to determine whether maximal IFNα release is optimal or instead is detrimental to anti-tumor responses. We chose five of these clones (with secretion rates of 200, 2,000 to 5,000, 18,000 to 25,000, 62,500, and 175,000 units/million cells/day - referred to here as MSC/a, MSC/b, MSC/c, MSC/d and MSC/e, respectively) to inject into the tail vein of C57Bl/6 mice injected subcutaneously in the right flank with 100,000 B16 cells. Tumor growth was monitored for 5 weeks and the rostrocaudal and dorsoventral tumor diameters were measured on days 27, 29, 32, 34, and 36 (depth diameter was assumed to be the mean of the other two diameters). The details of this experiment are described in Supplementary Text S3 in Additional file 3. Briefly, higher interferon doses inhibited initial tumor growth but actually promoted faster tumor growth, while lower doses of interferon within tumors did not prevent the initial growth of tumors but slowed their overall growth rate (Supplementary Figure S5 in Additional file 8).

## Conclusions

The use of adult-derived stem cells such as MSCs to modify the growth or properties of tissues is an active area of biomedical research. Expressing heterologous genes from MSCs will greatly expand the potential of MSCs in disease treatment. There is a bewildering variety of plasmids available for use, but generally each plasmid was designed for a particular purpose. Because the delivery of a given therapeutic should be controllable physiologically, an optimal vector for use in stem cell therapy should allow one to easily exchange various genetic elements to suit physiological needs. In the present manuscript we introduce a vector as well as a series of derivatives that are based on the well-validated plasmid pcDNA3. By inserting a few point mutations that insert useful restriction endonuclease sites, this plasmid (pc3.5) allows easy exchange of promoters and polyadenylation sequences controlling either the gene of interest or the antibiotic resistance gene, as well as the antibiotic resistance gene itself. Each of the derivative plasmids we synthesized resulted in both transient and stable expression of the GOI. The transfection efficiency as well as expression from these vectors is comparable to that seen for the most popular or effective vectors currently in use. In principle, viral vectors - which often generate engineered cells more efficiently than do plasmid vectors - can be similarly modified with a few nucleotide substitutions at borders between genetic elements to produce multifaceted viral vectors. However, each variant of a viral vector needs to be tested for viral packaging, infectivity and biosafety, which is a hindrance relative to plasmid-based vectors.

To visualize MSCs that express type I interferons, we constructed a bicistronic message in which the Mu-IFNαA cDNA is the first cistron while the ChFP cDNA is second cistron, whose translation was driven by the IRES from the EMCV genome. We ran into issues, however, trying to express this bicistronic message efficiently in MSCs using our initial vector pEF3. These issues were particularly obvious when comparing its expression with that of a plasmid that is provided as a standard in the Amaxa/Lonza Inc. nucleofection kit (Figures 5 to 7). Considerable improvements in the expression of bicistronic messages were discovered while resolving this issue. Although some improvement in the expression of the GOI came from modifying the expressing the puromycin instead of hygromycin or neomycin/geneticin) resistance genes (Figure 6), a greater improvement came from utilizing a stronger promoter and employing a chimeric 5' intron (Figure 7).

During the optimization process, we placed this bicistronic cDNA into a series of vectors expressing various promoters or eukaryotic antibiotic resistance genes (Figure 1). We successfully isolated clonal MSC lines from populations transfected with nearly all of these vectors. In these clonal cell lines, the cherry fluorescence of these cells was proportional to the rate of secretion of bioactive Mu-IFNαA from these cell lines (data not shown). Not surprisingly, Mu-IFNαA secretion was generally weaker from clones expressing bicistronic messages than from clones expressing only Mu-IFNαA. In most clones secreting more than 5,000 units of interferon per 1 million cells per day, however, the specific activity of the released Mu-IFNαA in the conditioned medium ranged from 3 × 10^7 ^to 8 × 10^7 ^units/mg whether the (intron-truncated) EF1A promoter, the 5' trimmed EF1A promoter, or the PGK-intron promoter was used to express the bicistronic cDNA (data not shown).

We noticed that the bicistronic MuIFNαAEMCVChFP message transfected more poorly than the bicistronic EGFPEMCVChFP message in B16 cells and especially in MSCs (Figure 6). We believe this inhibition is due to a species-specific autocrine signaling loop of the synthesized Mu-IFNαA with the murine IFNα receptor present in murine MSCs. This is supported by the following observations: the inhibition of transfection or expression is not observed in human 293T cells (Figure 5); exogenous addition of Mu-IFNαA to B16 cells transfected with bicistronic messages slightly inhibited the transfection or expression of bicistronic messages that do not encode Mu-IFNαA (Figure 8); and the addition of neutralizing antibodies raised against Mu-IFNα slightly enhanced the expression of bicistronic messages encoding Mu-IFNαA in murine cells (Supplementary Figure S4 in Additional file 7). Notably, inhibition or enhancement of expression with exogenous treatment was not as effective as the decrease seen when Mu-IFNαA is expressed directly. Because vector-derived Mu-IFNαA is synthesized luminally prior to its secretion, while our Mu-IFNαA and monoclonal antibody treatments were extracellular, our failure to fully restore or inhibit the translation of various bicistronic messages could be explained by intraluminal signaling by high concentrations of newly-synthesized type I interferon within secretory vesicles. Hypothesizing that luminal signaling of newly synthesized type I interferons in MSCs greatly affects the expression of Mu-IFNαA-containing messages, this inhibition may be offset by inhibiting the expression or activity of type I interferon receptor though which Mu-IFNαA signals.

Related to this were issues with isolating clonal MSC lines expressing MuIFNαAEMCVChFP; these clones often grew slower than untransfected MSCs and were diluted out during the selection process and expansion of surviving clones. This is exemplified in Supplementary Figure S6 in Additional File 9, in which stably transfected cells expressing the bicistronic message MuIFNαAEMCVChFP only divided a few times relative to its neighbors that did not express this message (Supplementary Figure S6, top). By comparison, stably transfected MSCs expressing EGFPEMCVChFP (Supplementary Figure S6, middle in Additional file 9) and expressing TurboGFP from a monocistronic message (Supplementary Figure S6, bottom in Additional file 9) divided quickly, at a rate comparable with that for untransfected cells. Nevertheless, these stably transfected MSC lines appear to divide and have morphology resembling parental MSCs.

Paradoxically, higher doses of interferon within tumors prevented initial growth but accelerated the tumor growth rate (Supplementary Figure S5 in Additional file 8). This is rationalized based on the known anti-angiogenic activities of type I interferon [15], as angiogenesis is a prerequisite for tumor growth. Perhaps interferon should be present in higher doses during the establishment of tumors but be present only at lower doses once a tumor has been established. In agreement with this, it has been observed that cells chronically exposed to IFNα eventually lose sensitivity to IFNα [56]. Perhaps the high dosages of IFN are desensitizing tumors cells in a similar way, removing its beneficial effects, and the accelerated tumor growth rate is simply a less inhibited tumor doubling rate. If so, then use of an interferon with less potency - such as human IFNα1 [57,58] or type III interferon [11,56,59] - may be useful in the treatment of established epithelial tumors. The failure of IFNαA to inhibit tumor growth in our mouse models reinforces a need to consider alternate biotherapeutic strategies, such as TNFα or IL-24 that are known to have anti-angiogenic properties [60]. They could be used instead of IFNα or (with a second IRES) in addition to IFNα. Also, because certain therapeutics may be more effective against certain types of tumors and less effective in others, use of other tumor models or alternate strains of mice could help define the spectrum of effectiveness of various anti-tumor therapeutics. Finally, because each anti-tumor therapeutic may act by distinct mechanisms, use of mouse strains lacking various protein components (that is, recombinant immune receptors, immunocyte enzymes, cytokine receptors) can help delineate how each therapeutic exhibits its activity. Co-culture systems can also be used to investigate the molecular details of various anti-tumor mechanisms. Further studies are planned to utilize alternate therapeutics, alternate tumor models and alternate strains of wildtype or mutant mice.

Overall, we successfully used pc3.5-based plasmids and their derivatives to optimize both transient and stable transfections of plasmids in a variety of cell lines. We can easily exchange antibiotic resistance elements as well as mRNA-controlling elements. This plasmid family has been used for a variety of other purposes beyond the scope of the present manuscript. These plasmids can be used, coupled with an IRES, to make MSCs capable of being fluorescent and simultaneously secreting type I interferon, whose inhibitory effects on tumor growth may be at least partially distinct from its effects on inhibiting tumor establishment. These engineered MSCs can be visualized, and in this way are easily distinguished from parental or endogenous MSCs, and will help provide a real-time readout of the efficiency of engineered MSCs within tumors.

## Abbreviations

ChFP: monomeric cherry fluorescent protein; CMV: cytomegalovirus; DMEM: Dulbecco's modified Eagle's medium; EF1A: elongation factor-1α; ELISA: enzyme-linked immunosorbent assay; EMCV: encephalomyocarditis virus; FCS: fetal calf serum; GFP: monomeric green fluorescent protein; GOI: gene of interest; IFN: interferon; IL: interleukin; IRES: internal ribosome entry site; MSC: mesenchymal stem cell; ORF: open reading frame; PAGE: polyacrylamide gel electrophoresis; PBS: phosphate-buffered saline; PCR: polymerase chain reaction; PEI: polyethyleneimine; Pfu: *Pyrococcus furiosus*; PGK: phosphoglycerate kinase; SV40: simian virus-40; Taq: *Thermus aquaticus*; UTR: untranslated region.

## Competing interests

The authors declare that they have no competing interests.

## Authors' contributions

CDK designed plasmid pc3.5 and its derivatives, performed nearly all PCR amplifications, performed all fluorescence-activated cell sorting analyses, performed nearly all microscopy, and assembled the manuscript and the figures. LSI synthesized most of the pc3.5 derivatives, performed nearly all transient and stable transfections, carried all cell lines, and performed all interferon assays. GR and Z-RY performed the first experiments utilizing MSCs in mice. YS is the principal investigator of the laboratory of GR and Z-RY, assisted in the conception of the study, and advised GR and Z-RY on the initial mouse experiments. C-CC and YR advised and performed the second experiment in mice. SP conceived the study, supervised the submission of the manuscript, and is the principal investigator of the laboratory of CDK and LSI.

## Supplementary Material

Additional file 1 **Supplementary Text S1: Supplementary text with references**. Adobe PDF file containing information about how various PCR products were amplified. Click here for file

Additional file 2 **Supplementary Text S2: Supplementary text with references**. Adobe PDF file containing information about how various plasmids were synthesized. Click here for file

Additional file 3 **Supplementary Text S3: Supplementary text with references**. Adobe PDF file containing text describing the two mouse experiments. Click here for file

Additional file 4 **Supplementary Figure S1: The initial mouse experiment**. Adobe PDF file detailing the first mouse experiment. Reduced growth of tumors by MSCs ectopically expressing Mu-IFNαA. Plasmid pEF3-MuIFNαA was stably transfected into MSCs and a representative clone known to release high levels of fully bioactive Mu-IFNαA was amplified (MSC/IFNα). Fifteen mice were subdivided into five experimental groups of three mice each: in three mice, only B16 cells were injected (black); in three mice, B16 cells and parental MSCs were injected (red); three mice received B16 cells and MSC/IFNα cells (green); the next group of three mice received MSC/IFNα only after tumors derived from B16 cells were palpable, starting at day 7 (blue); the final three mice received only MSC/IFNα cells (purple). The dates of injection of B16 cells (black arrow) and of MSCs (green and blue arrows for zero and seven day injections, respectively) are labeled. **(a) **The maximum diameters of hind limbs at the site of tumor injection were measured for each mouse in the five groups at days 9 and 13 to gauge the rate of tumor growth. The diameters of each hind limb of each mouse in each group were averaged (*n *= 6, diamond) and the standard deviation of each group of data (vertical bars) were calculated. The lines between days 9 and 13 connect each group and imply the growth rate. **(b) **The numbers of mice surviving B16 tumor growth are reported. The colors of the lines correspond to the colors of the diamonds in (a). The black line was intentionally shifted down and to the left to illustrate the overlap with the red line. Click here for file

Additional file 5 **Supplementary Figure S2: Activity of the c-Myc internal ribosome entry site**. Adobe PDF file demonstrating unusual activity of the c-myc IRES. Varying efficiency of c-myc IRES-driven translation. Plasmid pEF3-ChFPcmycEGFP was transfected using PEI into 293T cells, and the transiently transfected population subjected to fluorescence-activated cell sorting. (Left) Intact cells were surrounded by the region labeled R1 on a forward scatter (inset, horizontal):side scatter (inset, vertical) contour plot. (Right) Disrupted cells were surrounded by the region labeled R1 on a forward scatter (inset, horizontal):side scatter (inset, vertical) contour plot. In both large figures, the R1-positively gated cells were then analyzed for the EGFP and ChFP fluorescence by a FL1 (main, horizontal) versus FL3 (main, vertical) contour plot. Nonfluorescent cells are surrounded by the R2-labeled polygon. Click here for file

Additional file 6 **Supplementary Figure S3: Analysis of pmaxGFP versus pmaxCDK-TurboGFP**. Adobe PDF file presenting a comparison of expression of two plasmids. Expression of copepod GFPs from pmax-based plasmids. Either PEI or Metafectene Easy (meta) were used to transfect 293T cells, B16 cells, or MSCs with pmaxCDK-TurboGFP (Tur, left-hand bars) or with pmaxGFP (ppl, right-hand bars). The upper bar graph displays the transfection efficiency, while the bottom bar graph displays the average FL1 fluorescence. It should be noted that, because the fluorescence of ppluGFP encoded within pmaxGFP is more yellowish than that of TurboGFP encoded within pmaxCDK, a larger fraction of ppluGFP fluorescence will pass through the FL1 barrier filter (515 to 545 nm) than that of TurboGFP fluorescence; this partially accounts for stronger FL1 fluorescence by pmaxGFP than by TurboGFP. Click here for file

Additional file 7 **Supplementary Figure S4: Apparent enhancement of transfections with monoclonal antibodies**. Adobe PDF file showing increased transfection and expression using monoclonal antibodies. Effect of neutralizing antibodies specific to Mu-IFNα on the transfection and expression of pCMVi.5puro-MuIFNαAEMCVChFP. B16 cells were transfected using Metafectene Easy with plasmid pCMVi.5puro-MuIFNαAEMCVChFP, and were either left untreated or were treated with neutralizing monoclonal antibodies raised against Mu-IFNα. (Left) The transfection efficiency (top) and average ChFP levels (bottom) were determined by fluorescence-activated cell sorting in the absence (black bars) and presence (gray bars) of the monoclonal antibodies. (Right) Contour plots of FL1 (horizontal) versus FL3 fluorescence are displayed of these cells. Untreated cells lie to the left, while antibody-treated cells lie to the right. Click here for file

Additional file 8 **Supplementary Figure S5: Interferon dose-dependant growth of mouse tumors**. Adobe PDF file demonstrating the second mouse experiment. Varying growth of B16 tumors in mice as a function of dosage release of MSCs. (Top) The growth rates of 100,000 injected B16 melanoma cells were monitored in three mice per group co-injected with monoclonal MSCs expressing Mu-IFNαA from the bicistronic message MuIFNαAEMCVChFP at the following doses (from left to right): 200, 2,000 to 5,000, 18,000 to 25,000, 62,500, or with a monoclonal MSCs expressing Mu-IFNαA from a monocistronic message at a dosage of 175,000 units/(10^6 ^cells/day). No growth is assumed if the tumor mass is below the limit of palpation (about 125 mm^3^). (Bottom) Assuming exponential growth of tumors within mice, best-fit lines of the exponential growth rate and initial tumor size were calculated on a semi-log plot (left). From these, values for initial tumor size and doubling rate can be obtained (right). Click here for file

Additional file 9 **Supplementary Figure S6: Images of stably transfected stem cells**. Adobe PDF file showing stable transfection of pPGK1.5hygro-based plasmids in MSCs. Transfections of pPGK1.5hygro-MuIFNαAEMCVChFP (top row), pPGK1.5hygro-EGFPEMCVChFP (middle row), and pPGK1.5hygro-TurboGFP (bottom panel) were done with Metafectene Easy in MSCs; and after treatment with hygromycin (100 μg/ml) for 2 weeks, cells were allowed to amplify to demonstrate their ability to grow as stem cells. (Top panel) The left image is that of red fluorescence; the middle image is that obtained with bright-field illumination. The right-hand image is a merge of the left-hand two images. The bright-field image was intentionally darkened to facilitate visualizing the overlap of the two images. (Middle panel) The left image is of red fluorescence; the right image is of green fluorescence. (Bottom panel) Green fluorescence of the image is shown. Click here for file
