# Supplementary material for: Efficient co-expression of bicistronic proteins in mesenchymal stem cells by development and optimization of a multifunctional plasmid
Source: Stem Cell Res Ther. 2011 Mar 14;2(2):15. doi: 10.1186/scrt56 (PMC3226286; doi:10.1186/scrt56)
Supplement: Additional file 1 — Supplementary Text S1: Supplementary text with references. Adobe PDF file containing information about how various PCR products were amplified. [file scrt56-S1.PDF]

SUPPLEMENTARY TEXT 1

The human elongation factor-1 (EF1A) promoter was amplified from plasmid pEF3 [29] with the following primers: ATATcccgggACGCGTGAGGCTCCGGTGCC (forward) and TGTGggatccGATCAAGCTTCACGACACCTGAAATGGAA (reverse). The *Mlu*I and *Hind*III sites used to subclone this fragment into pc3.5 are underlined. The *Sma*I and *BamH*I sites set in lowercase were used to subclone this promoter fragment into an unrelated vector.

The human cyclooxygenase-2 (COX2) promoter was amplified from human genomic DNA with the following primers: ATATcccgggACGCGTGCTGCATATAGAGCAG (forward) and TGTGggatccGATCAAGCTTTCGTATGACAATTGGTCG (reverse). The *Mlu*I and *Hind*III sites used to subclone this fragment into pc3.5 are underlined. The *Sma*I and *BamH*I sites set in lowercase were used to subclone this promoter fragment into an unrelated vector.

The human phosphoglycerate kinase-1 (PGK1) promoter was amplified from human genomic DNA with the following primers: CCTCACGCGTCACGGGGTTGGGGTTGCGC (forward) and ACAGCCCGGGAGAGAGGTCGGTGATT (reverse). The *Mlu*I and *SmaI/Xma*I sites used to subclone this fragment into pc3.5hygro at two different locations are underlined.

The human phosphoglycerate kinase-1 (PGK1) 3' untranslated region (UTR) was amplified from human genomic DNA with the following primers: CAATATCGATTACTTTCCTGCCTTTTAG (forward) and CTCAGTATACCTAACAAAGTATGACAGG (reverse). The *Cla*I and *BstZ17*I sites used to subclone this fragment into pc3.5 are underlined.

A -globin/IgG chimeric intron was amplified from plasmid pmaxGFP (Amaxa/Lonza, Inc., Gaithersburg, MD USA) with the following primers: TTGACTTAAGACAGGTAAGTATCAAGGTT (forward) and AACCAAGCTTACCCTGTGGAGAGAAAGGCAA (reverse). The *Afl*II and *Hind*III sites used to subclone this fragment into pc3.5hygro and pc3.5puro are underlined. An alternative forward

primer TTGAGTTAACACAGGTAAGTATCAAGGTT containing an underlined *Hpa*I site was used to subclone the intron into pc3.5.

The IRES from EMCV was amplified from two separate plasmids: ptet-tta5 and pIRES-EGFP (a gift from Jerome Langer, RWJMS-UMDNJ) with the following primers: ATAAGAATTCgatatcGCCCCTCTCCCTCCCCCC (forward) and either TAACGGCGCCGCGCATGGTACCgatatcTTGTGGCCATATTATCATC (reverse for ptet-tta5) or TAACGGCGCCGCGCATCGTACGgatatcTTGTGGCCATATTATCATC (reverse for pIRES-EGFP). The *EcoR*I and either *Kpn*I or *BsiW*I sites used to subclone this fragment into pEF3 are underlined. The *EcoR*V sites included in these primers are set in lowercase.

The IRES from the human c-myc P2 mRNA was amplified from human genomic DNA with the following primers: ATAAGAATTCgatatcAACTCGCTGTAGTAATTC (forward) and TAACGGCGCCGCGCATGGTACCgatatcCCAGCGTCTAAGCAGCTGC (reverse). The *EcoR*I and *Kpn*I sites used to subclone this fragment into pEF3 are underlined. The *EcoR*V sites included in these primers are set in lowercase.

The Mu-IFN- A (whose gene is the closest murine Type I IFN gene to the murine IFN- locus) open reading frame was amplified from plasmid pLNCX-Mu-IFN- A [10] with the following primers: GAATGGATCCATGGCTAGGCTCTGTGCTT (forward) and AGGTGATATCGAATTCTCACTCCTTCTCCTCACTC (reverse). The *BamH*I, *EcoR*I and *EcoR*V sites used to subclone this fragment into pEF3 are underlined.

The hygromycin open reading frame was amplified from plasmid 2-hTERT-hygro (a gift from Rick Cohen, Rutgers Stem Cell Research Center) with the following primers: CAAGCCCGGGATGAAAAAGCCTGAACTC (forward) and TTCCATCGATTTACCGATATTCCTTTGCCCTC (reverse). The *Xma*I and *Cla*I sites used to subclone

this fragment into pc3.5 are underlined.

The puromycin open reading frame was amplified from plasmid pPGK-Puro (a gift from Marc Brenneman, Rutgers University) with the following primers: ACTGCCCGGGACCATGACCGAGTACAAG (forward) and GACAATCGATTCAGGCACCGGGCTTGCG (reverse). The *Xma*I and *Cla*I sites used to subclone this fragment into pc3.5 are underlined. The PCR product that was integrated into pc3.5puro and its descendants has only a single silent mutation that fortuitously eliminated a *BssH*II site (underlined, **GAG**CGCGC -> **GAA**CGCGC, and resulted in a codon (in boldface) that is predicted to equally translated in mammalian cell lines as the original.

The TurboGFP open reading frame was first amplified from plasmid pGIPZ (a gift from Paul Copeland, RWJMS-UMDNJ) with the following primers: ACTCGGTACCAGGATCCACCATGCCACCATGGAGAGCGACG (forward) and GAATCTCGAGTGCGGCCGCGGATCCTTATTCTTCACCGGCATC (reverse). The *Kpn*I, *BamH*I, *Not*I, and *Xho*I sites used to subclone this fragment into pmaxGFP are underlined. After realizing that a premature out-of-frame start codon greatly reduced the translational efficiency of TurboGFP in this intermediate plasmid pCDK-TurboGFP (data not shown), we amplified TurboGFP with the above-mentioned reverse primer and this forward primer ACTCGGATCCAGGTACCGCCACCATGGAGAGCGAC, where the *BamH*I and *Kpn*I sites are underlined. This PCR product was subcloned into pCDK-TurboGFP, pCMVi.5hygro, pCMVi.5puro, pPGK1.5hygro, pPGK1.5puro, pPGKi.5hygro, or pPGKi.5puro.
