# Supplementary material for: Efficient co-expression of bicistronic proteins in mesenchymal stem cells by development and optimization of a multifunctional plasmid
Source: Stem Cell Res Ther. 2011 Mar 14;2(2):15. doi: 10.1186/scrt56 (PMC3226286; doi:10.1186/scrt56)
Supplement: Additional file 2 — Supplementary Text S2: Supplementary text with references. Adobe PDF file containing information about how various plasmids were synthesized. [file scrt56-S2.PDF]

SUPPLEMENTARY TEXT 2

The monocistronic vectors pEF3-Mu-IFN- A, pEF3-EGFP.2 and pEF3-ChFP.2 were made by digesting pEF3 with *BamH*I and *EcoR*I and discarding a small fragment of the multicloning site. The green and cherry fluorescent protein (EGFP, ChFP) open reading frames were isolated from plasmids pcDNA3-FL R1/EGFP or pcDNA3-FL R2/EGFP[A206K] by digestion with *BamH*I and *EcoR*I, and isolation of an approximately 720 bp fragment whose sequence is: GGATCCCATGGTGAGC {EGFP/ChFP ORF}TACAAGTAAGAATTC (the *BamH*I and *EcoR*I sites are underlined). The PCR encoding Mu-IFN- A was similarly digested and an approximately 600 bp piece whose sequence is GGATCCATGGCTAGG{Mu-IFN- A ORF}AAGGAGTGAGAATTC was isolated.

The bicistronic vectors pEF3-MuIFN AEMCVChFP, pEF3-EGFPEMCVChFP, pEF3-ChFPEMCVEGFP, pEF3-MuIFN AcmycChFP, pEF3-EGFPcmycChFP, and pEF3-ChFPcmycEGFP (Where the first and second cistrons are indicated by what phrase is before and after the words EMCV or cmyc respectively) were made by four-piece ligations. Plasmid pEF3 was digested with *BamH*I and *Not*I, and a small segment of the multicloning site discarded. The ORF encoding first cistron was digested with *BamH*I and *EcoR*I. The ORF encoding the second cistron was digested with either *Kpn*I or *Acc65*I (5' end) and *Not*I (3' end) from plasmids pEF3-EGFP or pEF3-ChFP, and the fragment of about 750 bp retained whose sequence was GGTACCATGGTGAGC{EGFP/ChFP ORF}TACAAGTAAGGATCC{multicloning site from pEF3/pcDNA3}GCGGCCGC. The PCR fragment encoding the c-myc IRES was digested with *EcoR*I and *Kpn*I, and the approximately 390 bp fragment whose sequence is GAATTCGATATCAACTCGCTG{IRES}AGACGCTGGGATATCGGTACC was isolated. The PCR fragment encoding the EMCV IRES from plasmid ptet-tta5 was digested with *EcoR*I and *Kpn*I, and the approximately 610 bp fragment whose sequence is GAATTCGATATCGCCCCTCTC{IRES}TGGCCACAAGATATCGGTACC was isolated. The PCR

fragment encoding the EMCV IRES from plasmid pIRES-EGFP was digested with *EcoR*I and *BsiW*I, and the approximately 610 bp fragment whose sequence is GAATTCGATATCGCCCCTCTC{IRES}TGGCCACAAGATATCCGTAGC was isolated. Here, a *BsiW*I was employed because this IRES has an endogenous *Kpn*I site was that mutated in ptet-tta5 (see below) whose presence is mandatory under our conditions for efficient IRES-driven translation. Correct ligation proceeded in this order: [plasmid-*BamH*I-first ORF-*EcoR*I-IRES-*Kpn*I-second ORF-*Not*I-plasmid] or [plasmid-*BamH*I-first ORF-*EcoR*I-IRES-*BsiW*I/*Acc65*I-second ORF-*Not*I-plasmid].

In most cases, the three bicistronic inserts containing the EMCV IRES were migrated among vectors by digestion of the inserts with *BamH*I, digestion of vectors with *BamH*I and processing with calf alkaline phosphatase, and ligation of cohesive ends. Correct orientation of inserts was confirmed by digest of DNA from well isolated and amplified colonies with either *Kpn*I or *Acc65*I.

To place these bicistronic inserts under the control of the EF3.5b promoter, a two-step procedure was followed. First, the insert was first placed into intermediate plasmid pZeroTG-EF35b. Next, the promoter/insert complex was removed from its 3.6 kb pZeroTG vector backbone (a gift from Kristoffer Valerie, Virginia Commonwealth University) by digestion with *Mlu*I and *Not*I; this piece was moved to pc3.5-based vectors where the antibiotic gene elements were modified (see below). Vectors pc3.5PGKhygro, pc3.5puro, and pc3.5neoPGK, were digested with *Mlu*I and *Not*I, and the smaller fragment encoding the CMV promoter and part of the multicloning site discarded. Ligation of cohesive ends yielded pEF3.5bPGKhygro-(insert), pEF3.5puro-(insert) and pEF3.5bneoPGK-(insert).

Several vectors were constructed with various promoters substituting the CMV promoter in pc3.5. PCR products of the EF1A and COX2 promoters were digested with *Mlu*I and *Hind*III and the fragments of 839 and 624 bp retained. Plasmid pc3.5 was digested with *Mlu*I and *Hind*III, and the small fragment encoding the CMV promoter discarded. Ligation of cohesive ends yielded plasmids pEF3.5b and

pCOX2.5. The ‘b’ is used because we created two shorter promoter PCR products (46 and 86 bps truncated at the 5' end) that gave suboptimal expression (approximately 80 and 60% that of pEF3 respectively) of various fluorescent proteins. We amplified the promoter from plasmid pEF3 by PCR instead of amplifying the promoter from the human genome or excising the promoter directly from pEF3 in order to (1) avoid 127 nucleotides after the *Mlu*I site of pEF3 that derived from part of the 5'UTR and open reading frame of the T-antigen gene from the SV40 genome that also contains a *Hind*III site (all from the pEF-BOS vector that was the original source of the EF1A promoter [42]), and (2) to work with the intron-truncated promoter that was 353 bp shorter than the wild-type promoter and retained full promoter activity [29,50]. A schematic of the design of truncated EF1A constructs is shown in Fig. 6.

The human PGK1 promoter was placed upstream of both the multicloning site and the antibiotic resistance gene in separate vectors. The PCR product encoding the PGK1 promoter was digested with Either *Sma*I or *Xma*I (depending on which vector was utilized) and *Mlu*I, and a fragment of about 510 bp isolated. If the PGK1 promoter is used to express genes placed within the multicloning site, plasmid pc3.5hygro or plasmid pc3.5puro was digested with *Mlu*I and *Hpa*I and the small fragment encoding the CMV promoter discarded. Ligation of cohesive ends (and fusion of blunt ends) yielded pPGK1.5hygro and pPGK1.5puro. Note that, with these constructs, the T7 primer site remains. To instead drive the antibiotic resistance gene, plasmid pc3.5hygro (whose *BssH*II site is unique) was digested with *BssH*II and *Xma*I, discarding the small fragment encoding the SV40 promoter SV40 replication origin, and T-antigen binding site. Ligation of cohesive ends (*Mlu*I to *BssH*II and *Xma*I to itself) yielded plasmid pc3.5PGKhygro.

The CMV and PGK promoters above do not contain introns and thus drive intronless mRNA expression. The chimeric intron was inserted into plasmids pc3.5hygro, pc3.5puro, pPGK1.5hygro, pPGK1.5puro to test whether forcing RNA processing of a GOI will improve the expression of CMV-

driven or PGK-driven inserts in our system.

Plasmids pc3.5hygro and pc3.5puro were digested with *Afl*II and *Hind*III, and a very small fragment encoding the T7 primer site was discarded. The intron PCR product that was generated with primers producing *Afl*II and *Hind*III ends were digested with the same enzyme pair. Ligation of cohesive ends yielded plasmids pCMVihygro and pCMVipuro, where CMVi indicates the CMV promoter with the included intron. The chimeric intron was placed upstream of the PGK-1 promoter in a three-piece ligation. The chimeric intron PCR fragment with *Hpa*I and *Hind*III ends was digested with *Hpa*I and *Hind*III. Plasmids pc3.5hygro and pc3.5puro were digested with *Mlu*I and *Hind*III. With the PGK promoter fragment obtained above, ligation of cohesive ends yielded pPGKi.5hygro and pPGKi.5puro, where 'PGKi' denoted the human PGK-1 promoter with an included intron, by the ligation order: [vector-*Mlu*I-PGK promoter-*Hpa*I-intron-*Hind*III-vector].

As mentioned above, pCDK-TurboGFP was an intermediate plasmid. Inserts were placed into this plasmid by digesting the plasmid with *BamH*I, treating with shrimp alkaline phosphatase, and removing the 700 bp insert encoding the faulty TurboGFP insert. Bicistronic inserts already digested with *BamH*I above were ligated to this plasmid as described above to make plasmids pmaxCDK-MuIFN AEMCVChFP, pmaxCDK-EGFPEMCVChFP, and pmaxCDK-ChFPEMCVEGFP.

After the correct TurboGFP PCR product (with only one start codon) was integrated into the pCDK-TurboGFP vector (by ligation of *BamH*I-digested fragments), the resulting plasmid, pmaxCDK-TurboGFP, had the following sequence (starting from the *Kpn*I site and ending at the *Xho*I site that are part of pmaxCloning and pmaxGFP vectors: GGTACCAGGATCCAGGTACCGCCACC**ATG**GAGAGC{TurboGFP ORF}GAAGAA**TAA**GGATCCGCGGCCGCACTCGAG. This TurboGFP insert was subcloned into pCMVi.5hygro, pCMVi.5puro, pPGK1.5hygro, pPGK1.5puro, pPGKi.5hygro, pPGKi.5puro using ligation

of *BamH*I-digested fragments as described above for exchanging bicistronic insert among vectors.

The PCR products encoding hygromycin and puromycin, as well as plasmid pc3.5 were digested with *Xma*I and *Cla*I, and the large DNA fragments kept. Ligation of cohesive fragments yielded plasmids pc3.5hygro and pc3.5puro.

To insert the 3'UTR of the PGK-1 gene into plasmid pc3.5 so that it controls the antibiotic resistance gene, both the PCR product encoding the PGK-1 3'UTR as well as plasmid pc3.5 was digested with *Cla*I and *BstZ17*I, and the large DNA fragments kept. Ligation of cohesive ends yielded plasmid pc3.5neoPGK.

Alternatively, the entire antibiotic resistance gene in pc3.5 was exchanged so that no viral elements were included. Plasmid pc3.5PGKhygro was digested with *Xma*I and *BstZ17*I, and the smaller fragments encoding the hygromycin gene and the SV40 polyadenylation motif discarded. The puromycin cDNA and PGK1 3'UTR fragments described above were ligated to the vector in a three piece ligation to yield plasmid pc3.5PGKpuroPGK with the proper ligation order [vector-*Xma*I-puromycin-*Cla*I-PGK1UTR-*BstZ17*I-vector].
