# Supplementary material for: Efficient co-expression of bicistronic proteins in mesenchymal stem cells by development and optimization of a multifunctional plasmid
Source: Stem Cell Res Ther. 2011 Mar 14;2(2):15. doi: 10.1186/scrt56 (PMC3226286; doi:10.1186/scrt56)
Supplement: Additional file 3 — Supplementary Text S3: Supplementary text with references. Adobe PDF file containing text describing the two mouse experiments. [file scrt56-S3.PDF]

SUPPLEMENTARY TEXT 3

In our first mouse experiment, to test the hypothesis that MSCs expressing Mu-IFN- A inhibit tumor growth and development, we amplifed a representative clone secreting a high dose of Mu-IFN- A. These cells were injected to C57Bl/6 mice either concurrently with B16 melanoma cells (resembling scenarios in which tumor development is predicted clinically or where a primary but potentially metastatic tumor was excised, and subordinate suppressed tumors may remain) or after palpable tumors were detected (modeling a scenario where a tumor is detected but tumor excision is infeasible or is trying to be avoided), or was injected in the absence of B16 cells to ensure that these cells by themselves are not toxic to mice. As controls, B16 cells were injected in the absence of MSC to follow how fast unencumbered tumors grow in mice; also B16 cells were co-injected with untransfected MSCs to ensure that the benefit of MSCs requires IFN- A secretion.

Tumor development was followed 9 and 13 days after injection by measuring the maximum diameter of the hind limb at the site of tumor injection in each of the five groups of mice (Supplementary Fig. S1A in Additional file 4). Compared to mice receiving only B16 cells, only mice coinjected with B16 cells and MSC/IFN- ($p = 0.0007$) and mice injected with only MSC/IFN- ($p < 0.0001$) had statistically smaller tumor sizes on day 9. By day 13, mice coinjected with B16 cells and parental MSCs had noticeably but not statistically significant larger tumor size than mice injected with B16 cells alone ($p = 0.0928$); this may be due to increased immunosuppressive effects of MSCs on the anti-tumor immune system [5,6]. Not surprisingly, mice coinjected with B16 and MSC/IFN- maintained significantly smaller tumor size than either B16 cells or B16 cells coinjected with parental MSCs ($p < 0.0001$,0.0001). These mice also had statistically smaller tumor size than mice injected with MSC/IFN- after tumors were palpable ($p = 0.0024$). Tumor size on day 13 in mice injected with MSC/IFN- after tumors were palpable was noticably but not significantly smaller than those seen in mice injected with only B16 cells

(p = 0.1502), but was significantly smaller than those in mice coinjected with B16 calls and parental MSCs (p = 0.0081). This result suggested that IFN's major effects are early in tumor development. Finally, engineered MSCs do not by themselves affect the thickness of hind limbs.

Additionally, the date of death of each of the three mice in each group was noted (Supplementary Fig. S1B). The viability curves were consistent with the development of tumors, such that slower tumor growth correlated with longer viability. Mice injected with only tumor cells die almost as quickly as those injected with tumor cells and untransfected MSCs, with no statistical difference between the two viability curves (p = 0.91). Mice coinjected with engineered MSCs and tumor cells lived longer, attaining statistical significance (p = 0.025); mice injected with engineered MSCs only after tumors were palpable did live longer, but this difference did not achieve statistical significance (p = 0.063). Finally, mice injected only with engineered stem cells lived for weeks without any overt physiological consequences (p = 0.025). Injecting MSCs as early as possible was important for viability: statistical significance was achieved between B16 cells and MSC/IFN- injected at either day 0 or day 7 (p = 0.025).

In our second mouse experiment, we chose four MSC clones expressing the MuIFN AEMCVChFP mRNA (with secretion rates of 200, 2000-5000, 18000-25000, and 62500, referred to here as MSC/a, MSC/b, MSC/c, MSC/d respectively) and the MSC clone expressing the monocistronic MuIFN A mRNA with a secretion rate of 175000 units/million cells/day that we used in the experiment in Supplementary Figure S1 that we call MSC/e here. These five MSC clones were injected into the tail vein (500,000 in each of three mice in each group on days 0, 3, and 6) of C57Bl/6 mice injected subcutaneously in the right flank with 100,000 B16 cells. Tumor growth was monitored for 5 weeks, with rostrocaudal and dorsoventral tumor diameters measured on days 27, 29, 32, 34, and 36 (interior diameter was assumed to be the mean of the other two diameters). We found that none of the three mice transfected with MSC/e grew palpable tumors after 36 days, while only one, two, and one of

the three mice injected with MDC/d, MSC/c and MSC/b respectively grew tumors (Supplementary Fig. S5 in Additional file 8, top). Finally, all 3 mice injected with MSC/a grew tumors. One may preliminarily conclude that a sufficiently high dose of IFN- can effectively inhibit initial tumor growth.

In the mice harboring expanding tumors, tumor growth occurred at a nearly exponential rate; this was apparent as a qualitatively linear increase in semi-logarithmic tumor volume over time (Supplementary Fig. S5, bottom). Assuming an exponential growth rate of tumor volume, we determined the tumor doubling time (related to chronic tumor physiology) as well as an apparent initial tumor size from a straight line best-fit of the semi-log tumor volume growth kinetics (Supplementary Fig. S5, bottom right). We hypothesized that this analysis would separate the anti-tumor establishing and anti-tumor growth inhibiting effects of MSC-derived IFN- A.

Increasing intra-tumor doses of IFN- lowered the initial tumor volume in mice exhibiting tumors; we speculate that this effect is analogous to the inhibition of tumor establishment by high doses of IFN- in this experiment. In mice exhibiting tumors, the tumor growth rate generally increased with increasing IFN- release by the transfected MSCs. In mice injected with MSC/a (the lowest dose), the tumors grew relatively slowly, with doubling times of 3.1 d, 3.4 d, and 5.3 d for each of the tumors within the group. In mice injected with MSC/b, MSC/c, and MSC/d, the growth rates of tumors were 2.5 d for MSC/b, 2.0 d and 3.4 d for MSC/c, and 1.6 d for MSC/d. We are unsure why IFN- inhibits initial tumor establishment but favors chronic tumor growth under these conditions, nor are we sure why the doubling time is inversely proportional to the apparent initial size of the tumor.

Seven of eight mice that did not grow palpable tumors after 36 days were re-inoculated with either 500,000 or 1,000,000 B16 cells (five or ten times the initial dose) six days later to see whether presumably established MSCs prevent establishment of new tumors. Only one mouse (initially injected with MSC/d and later injected with 500,000 B16 cells) grew a tumor; this tumor was palpable only 21

days post-injection, and needed 20 more days to attain an approximate volume of 10^4 mm^3 (unpublished observations); this growth rate (with a tumor doubling time of 3-3.5 days) is comparable to the slower rate of tumor growth seen in mice injected with MSC/a. It is tempting to speculate whether adaptive immunity or innate antitumor mechanisms underlies the resistance of these mice to harboring these highly tumorigenic cell lines.
