# Supplementary figures and images for: Efficient co-expression of bicistronic proteins in mesenchymal stem cells by development and optimization of a multifunctional plasmid
Source: Stem Cell Res Ther. 2011 Mar 14;2(2):15. doi: 10.1186/scrt56 (PMC3226286; doi:10.1186/scrt56)

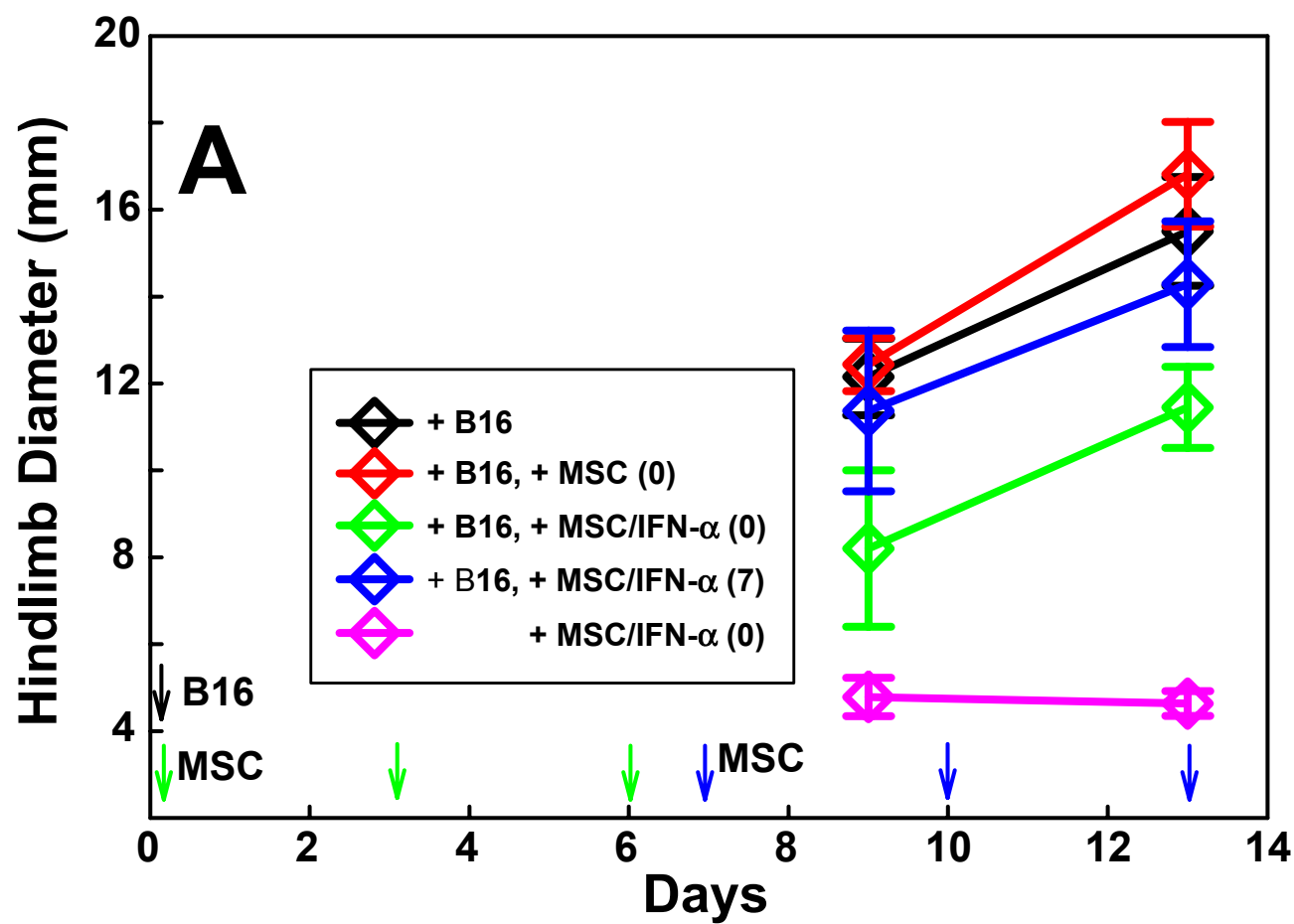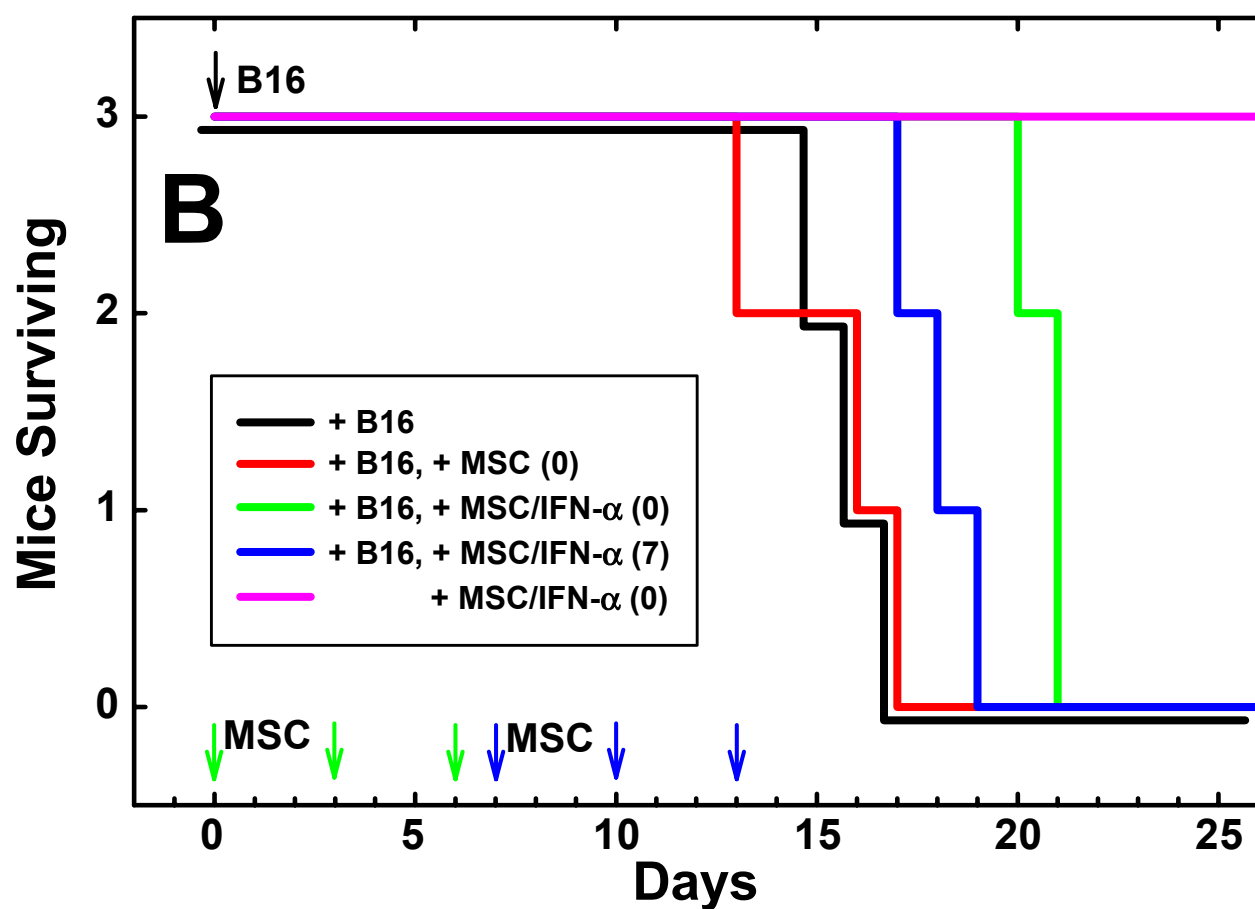

Supplementary Figure 1

Supplement: Additional file 4 — Supplementary Figure S1: The initial mouse experiment. Adobe PDF file detailing the first mouse experiment. Reduced growth of tumors by MSCs ectopically expressing Mu-IFNαA. Plasmid pEF3-MuIFNαA was stably transfected into MSCs and a representative clone known to release high levels of fully bioactive Mu-IFNαA was amplified (MSC/IFNα). Fifteen mice were subdivided into five experimental groups of three mice each: in three mice, only B16 cells were injected (black); in three mice, B16 cells and parental MSCs were injected (red); three mice received B16 cells and MSC/IFNα cells (green); the next group of three mice received MSC/IFNα only after tumors derived from B16 cells were palpable, starting at day 7 (blue); the final three mice received only MSC/IFNα cells (purple). The dates of injection of B16 cells (black arrow) and of MSCs (green and blue arrows for zero and seven day injections, respectively) are labeled. (a) The maximum diameters of hind limbs at the site of tumor injection were measured for each mouse in the five groups at days 9 and 13 to gauge the rate of tumor growth. The diameters of each hind limb of each mouse in each group were averaged (n = 6, diamond) and the standard deviation of each group of data (vertical bars) were calculated. The lines between days 9 and 13 connect each group and imply the growth rate. (b) The numbers of mice surviving B16 tumor growth are reported. The colors of the lines correspond to the colors of the diamonds in (a). The black line was intentionally shifted down and to the left to illustrate the overlap with the red line. [file scrt56-S4.PDF]

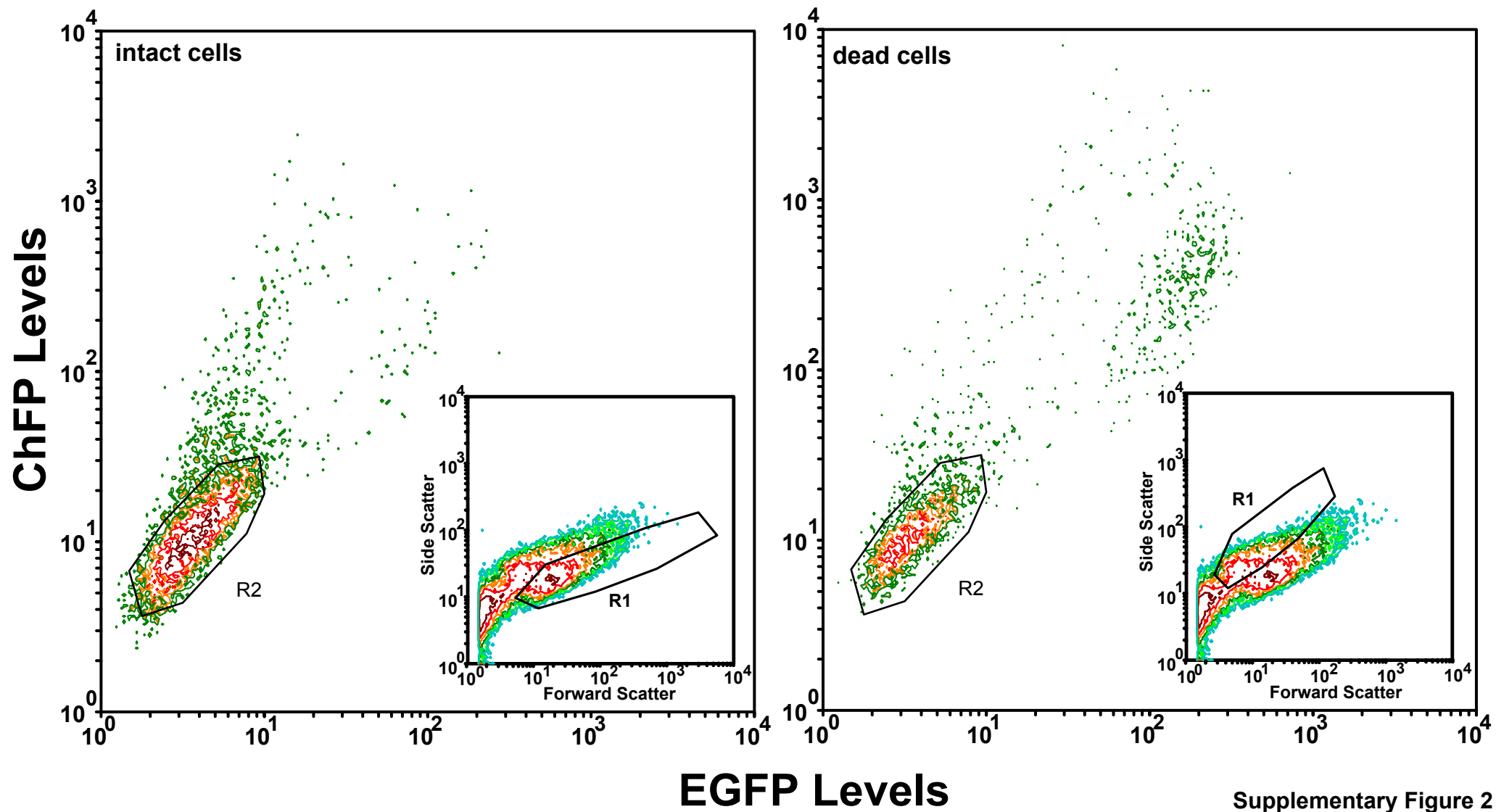

Supplement: Additional file 5 — Supplementary Figure S2: Activity of the c-Myc internal ribosome entry site. Adobe PDF file demonstrating unusual activity of the c-myc IRES. Varying efficiency of c-myc IRES-driven translation. Plasmid pEF3-ChFPcmycEGFP was transfected using PEI into 293T cells, and the transiently transfected population subjected to fluorescence-activated cell sorting. (Left) Intact cells were surrounded by the region labeled R1 on a forward scatter (inset, horizontal):side scatter (inset, vertical) contour plot. (Right) Disrupted cells were surrounded by the region labeled R1 on a forward scatter (inset, horizontal):side scatter (inset, vertical) contour plot. In both large figures, the R1-positively gated cells were then analyzed for the EGFP and ChFP fluorescence by a FL1 (main, horizontal) versus FL3 (main, vertical) contour plot. Nonfluorescent cells are surrounded by the R2-labeled polygon. [file scrt56-S5.PDF]

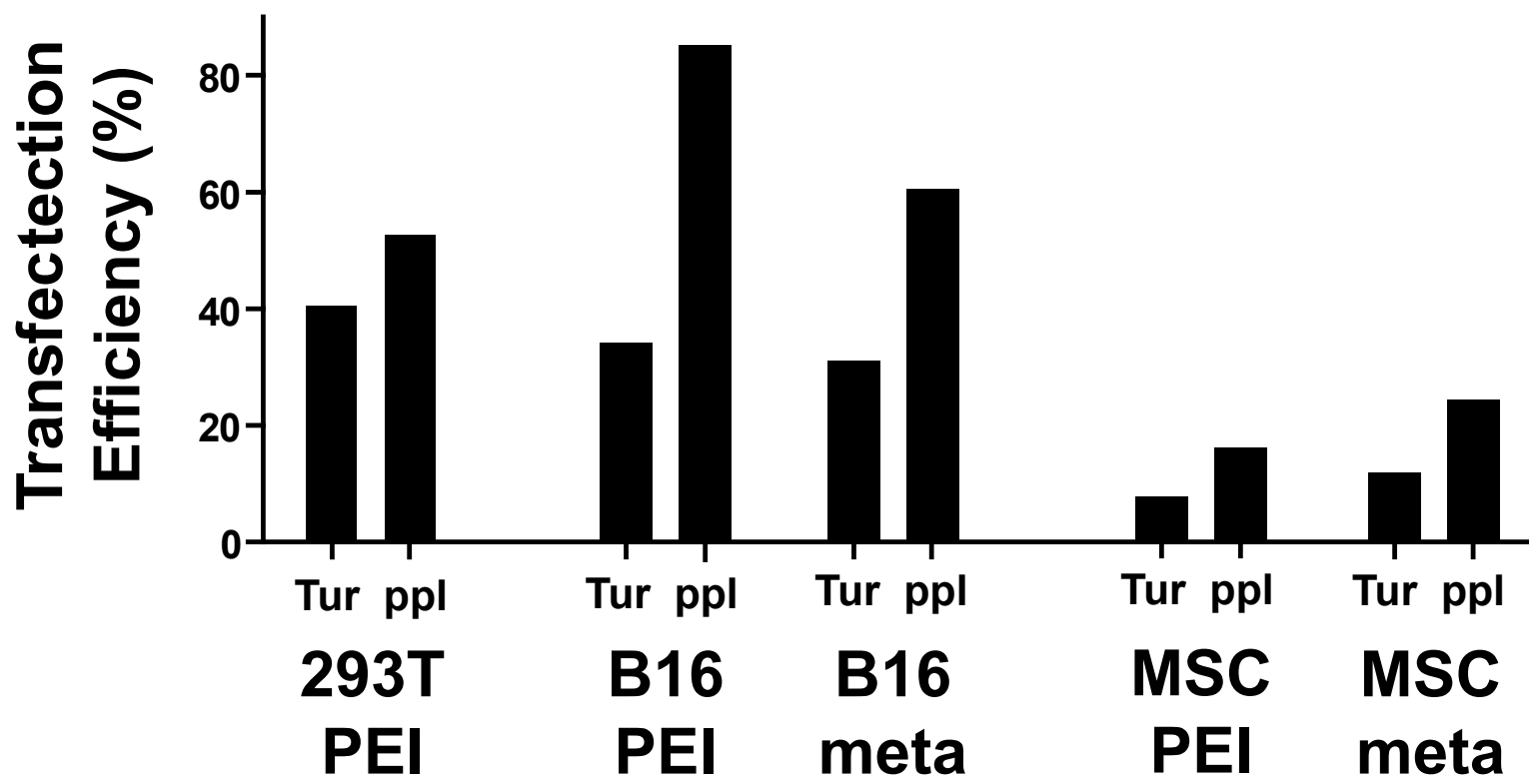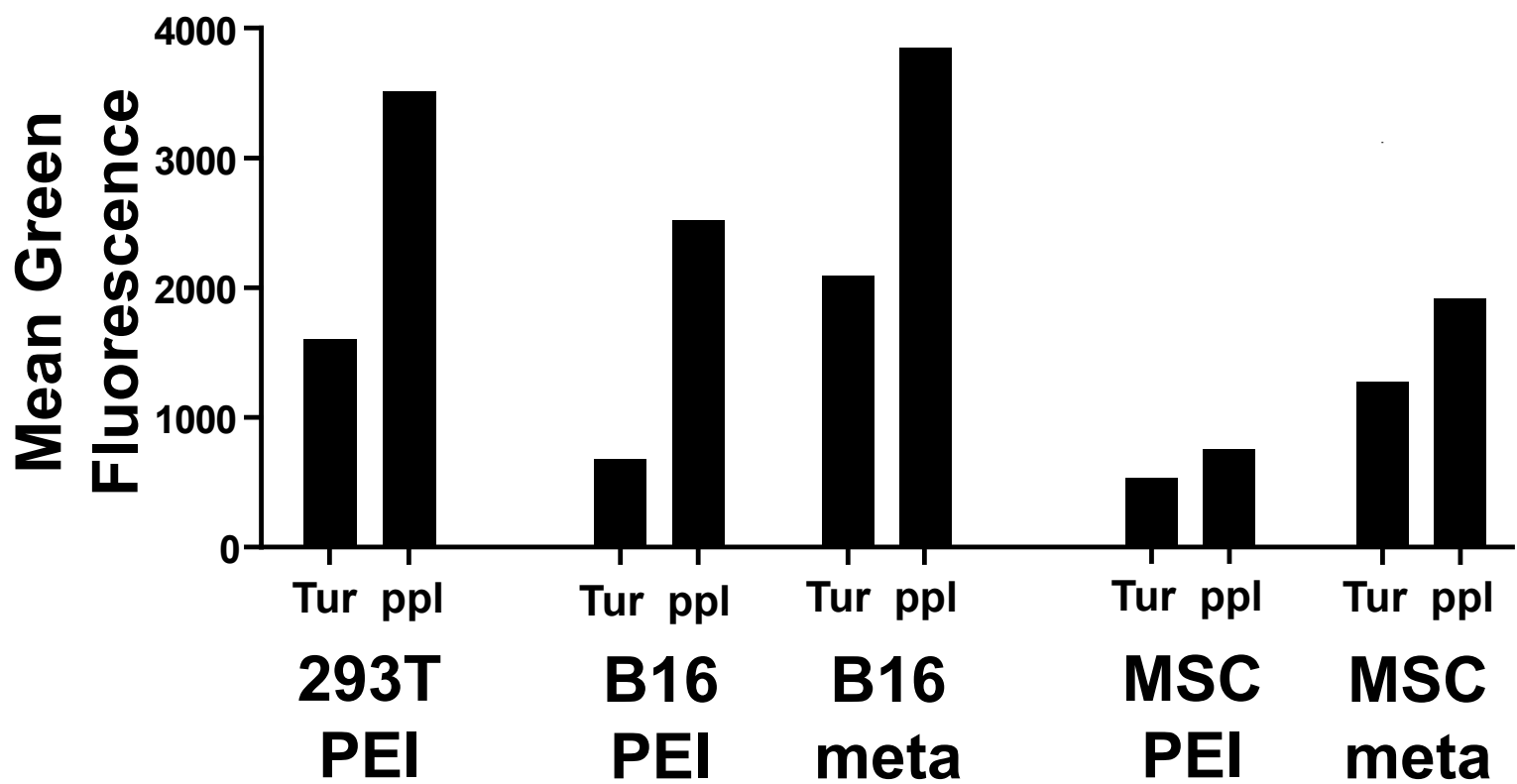

Supplement: Additional file 6 — Supplementary Figure S3: Analysis of pmaxGFP versus pmaxCDK-TurboGFP. Adobe PDF file presenting a comparison of expression of two plasmids. Expression of copepod GFPs from pmax-based plasmids. Either PEI or Metafectene Easy (meta) were used to transfect 293T cells, B16 cells, or MSCs with pmaxCDK-TurboGFP (Tur, left-hand bars) or with pmaxGFP (ppl, right-hand bars). The upper bar graph displays the transfection efficiency, while the bottom bar graph displays the average FL1 fluorescence. It should be noted that, because the fluorescence of ppluGFP encoded within pmaxGFP is more yellowish than that of TurboGFP encoded within pmaxCDK, a larger fraction of ppluGFP fluorescence will pass through the FL1 barrier filter (515 to 545 nm) than that of TurboGFP fluorescence; this partially accounts for stronger FL1 fluorescence by pmaxGFP than by TurboGFP. [file scrt56-S6.PDF]

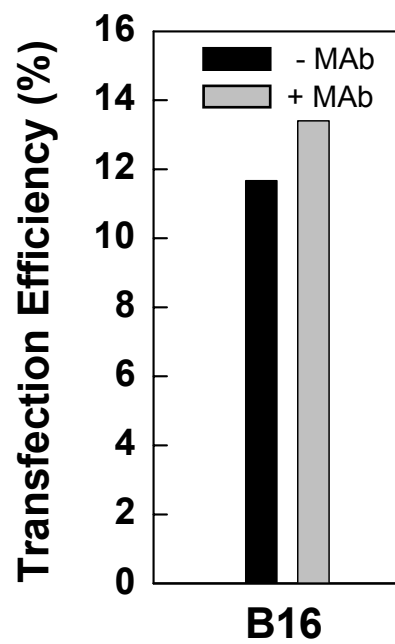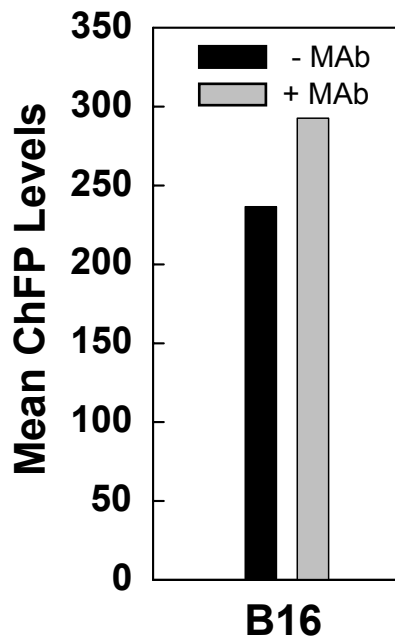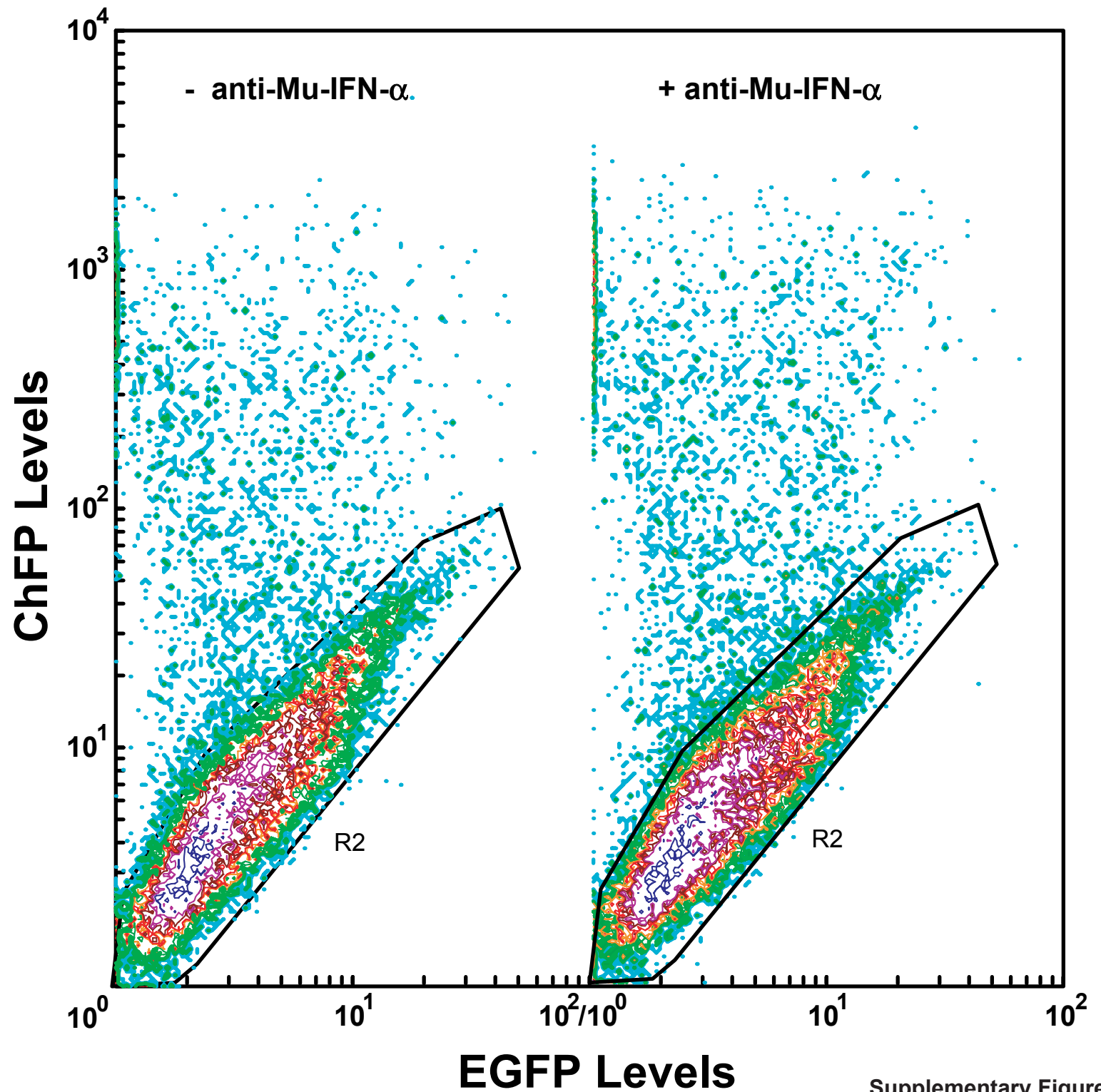

Supplementary Figure 4

Supplement: Additional file 7 — Supplementary Figure S4: Apparent enhancement of transfections with monoclonal antibodies. Adobe PDF file showing increased transfection and expression using monoclonal antibodies. Effect of neutralizing antibodies specific to Mu-IFNα on the transfection and expression of pCMVi.5puro-MuIFNαAEMCVChFP. B16 cells were transfected using Metafectene Easy with plasmid pCMVi.5puro-MuIFNαAEMCVChFP, and were either left untreated or were treated with neutralizing monoclonal antibodies raised against Mu-IFNα. (Left) The transfection efficiency (top) and average ChFP levels (bottom) were determined by fluorescence-activated cell sorting in the absence (black bars) and presence (gray bars) of the monoclonal antibodies. (Right) Contour plots of FL1 (horizontal) versus FL3 fluorescence are displayed of these cells. Untreated cells lie to the left, while antibody-treated cells lie to the right. [file scrt56-S7.PDF]
