# Supplementary material for: Efficient co-expression of bicistronic proteins in mesenchymal stem cells by development and optimization of a multifunctional plasmid
Source: Stem Cell Res Ther. 2011 Mar 14;2(2):15. doi: 10.1186/scrt56 (PMC3226286; doi:10.1186/scrt56)
Supplement: Additional file 8 — Supplementary Figure S5: Interferon dose-dependant growth of mouse tumors. Adobe PDF file demonstrating the second mouse experiment. Varying growth of B16 tumors in mice as a function of dosage release of MSCs. (Top) The growth rates of 100,000 injected B16 melanoma cells were monitored in three mice per group co-injected with monoclonal MSCs expressing Mu-IFNαA from the bicistronic message MuIFNαAEMCVChFP at the following doses (from left to right): 200, 2,000 to 5,000, 18,000 to 25,000, 62,500, or with a monoclonal MSCs expressing Mu-IFNαA from a monocistronic message at a dosage of 175,000 units/(106 cells/day). No growth is assumed if the tumor mass is below the limit of palpation (about 125 mm3). (Bottom) Assuming exponential growth of tumors within mice, best-fit lines of the exponential growth rate and initial tumor size were calculated on a semi-log plot (left). From these, values for initial tumor size and doubling rate can be obtained (right). [file scrt56-S8.PDF]

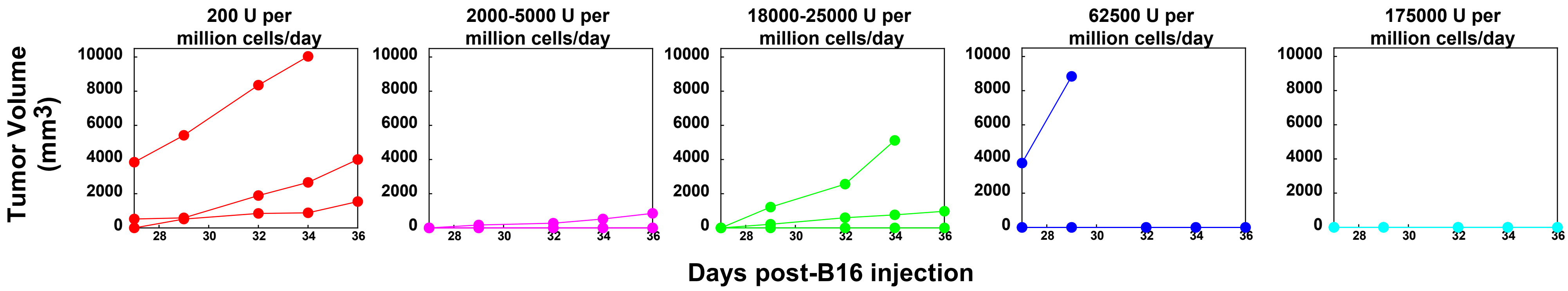

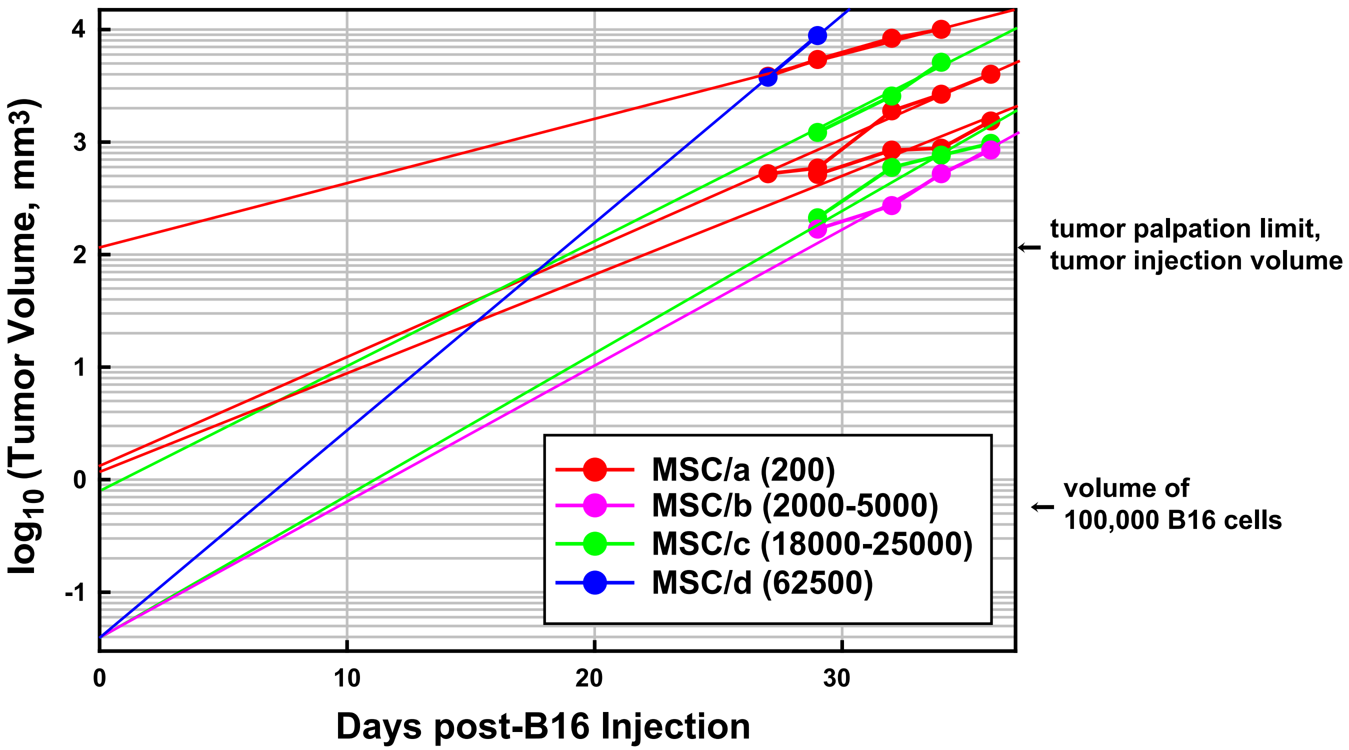

MSC dosage units ($\frac{\text{units}}{10^6\ \text{cells} \bullet \text{day}}$)	initial size (mm^3)	doubling time (days)
200	120	5.3
200	1.3	3.1
200	1.1	3.4
2000-5000	0.04	2.5
18000-	0.04	2.0
25000	0.76	3.4
62500	0.04	1.6

Supplementary Figure 5
