# Supplementary material for: Efficient co-expression of bicistronic proteins in mesenchymal stem cells by development and optimization of a multifunctional plasmid
Source: Stem Cell Res Ther. 2011 Mar 14;2(2):15. doi: 10.1186/scrt56 (PMC3226286; doi:10.1186/scrt56)
Supplement: Additional file 9 — Supplementary Figure S6: Images of stably transfected stem cells. Adobe PDF file showing stable transfection of pPGK1.5hygro-based plasmids in MSCs. Transfections of pPGK1.5hygro-MuIFNαAEMCVChFP (top row), pPGK1.5hygro-EGFPEMCVChFP (middle row), and pPGK1.5hygro-TurboGFP (bottom panel) were done with Metafectene Easy in MSCs; and after treatment with hygromycin (100 μg/ml) for 2 weeks, cells were allowed to amplify to demonstrate their ability to grow as stem cells. (Top panel) The left image is that of red fluorescence; the middle image is that obtained with bright-field illumination. The right-hand image is a merge of the left-hand two images. The bright-field image was intentionally darkened to facilitate visualizing the overlap of the two images. (Middle panel) The left image is of red fluorescence; the right image is of green fluorescence. (Bottom panel) Green fluorescence of the image is shown. [file scrt56-S9.PDF]

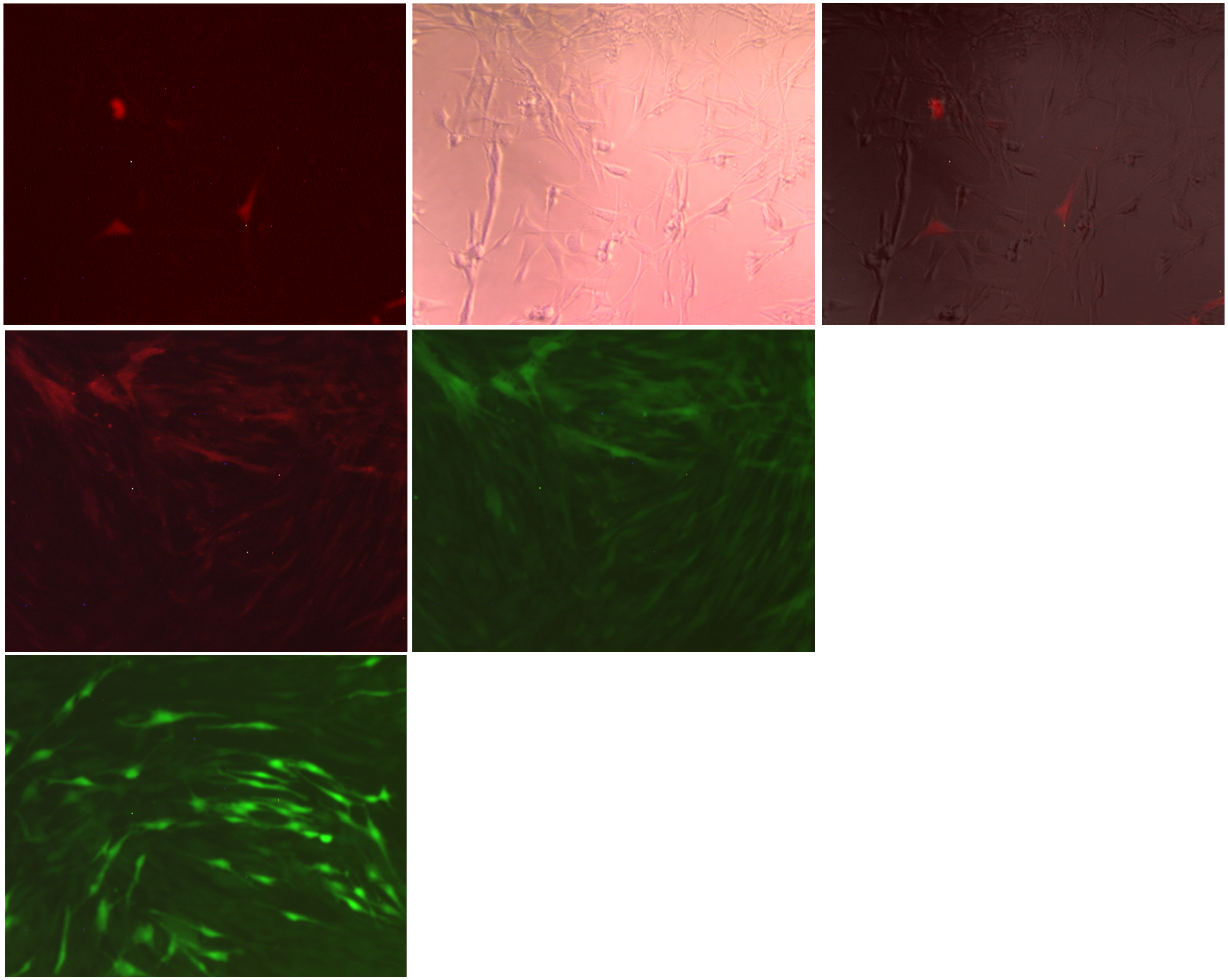

pPGK1.5hygro-MuIFNαAEMCVChFP

- Red Channel Illumination
- Brightfield Illumination
- Merge

pPGK1.5hygro-EGFPEMCVChFP

- Red Channel Illumination
- Green Channel Illumination

pPGK1.5hygro-TurboGFP

- Green Channel Illumination

Supplementary Figure 6
